# The Measurement of Maximal (Anaerobic) Power Output on a Cycle Ergometer: A Critical Review

**DOI:** 10.1155/2013/589361

**Published:** 2013-08-29

**Authors:** Tarak Driss, Henry Vandewalle

**Affiliations:** ^1^CeRSM, E.A. 2931, Equipe de Physiologie et de Biomécanique du Mouvement, UFR STAPS, Université Paris Ouest Nanterre—La Défense, 200 avenue de la République, 92000 Nanterre, France; ^2^Laboratoire de Physiologie, UFR de Santé, Médecine et Biologie Humaine, Université Paris XIII, Rue Marcel Cachin, 93017 Bobigny Cedex, France

## Abstract

The interests and limits of the different methods and protocols of maximal (anaerobic) power (*P*_max_) assessment are reviewed: single all-out tests *versus* force-velocity tests, isokinetic ergometers *versus* friction-loaded ergometers, measure of *P*_max_ during the acceleration phase or at peak velocity. The effects of training, athletic practice, diet and pharmacological substances upon the production of maximal mechanical power are not discussed in this review mainly focused on the technical (ergometer, crank length, toe clips), methodological (protocols) and biological factors (muscle volume, muscle fiber type, age, gender, growth, temperature, chronobiology and fatigue) limiting *P*_max_ in cycling. Although the validity of the Wingate test is questionable, a large part of the review is dedicated to this test which is currently the all-out cycling test the most often used. The biomechanical characteristics specific of maximal and high speed cycling, the bioenergetics of the all-out cycling exercises and the influence of biochemical factors (acidosis and alkalosis, phosphate ions…) are recalled at the beginning of the paper. The basic knowledge concerning the consequences of the force-velocity relationship upon power output, the biomechanics of sub-maximal cycling exercises and the study on the force-velocity relationship in cycling by Dickinson in 1928 are presented in Appendices.

## 1. Introduction

For a long time, the physical examination of athletes mainly consisted in the study of cardiovascular performances and endurance. Most researches were focused on the assessment of maximal oxygen uptake (*V*_O2_max⁡) and the power or velocity which corresponds to *V*_O2_max⁡ (maximal aerobic power or velocity). In the laboratory, these tests were performed on a treadmill or a cycle ergometer. Large scale studies were often carried out on friction-braked cycle ergometers such as Fleisch ergostat [1] and von Döbeln ergometer [2].

The pertinence of the assessment of these aerobic tests was highly debatable for the athletes who were specialised in power events (sprint, jumping, throwing, etc.) and performed short “supramaximal” exercises, that is, exercises whose power output was higher than the maximal aerobic power. Physical examination could not be restricted to aerobic testing but had to include the assessment of anaerobic performance. Moreover, it became obvious that the assessment of mechanical factors determining athletic performances (strength, speed, and maximal mechanical power) should be added to the usual tests mainly focused on bioenergetics. Maximal mechanical power was estimated from the results of vertical jump tests and staircase tests derived from the tests previously proposed [3–5]. The laboratories involved in physical examination generally possessed a friction-braked cycle ergometer and several tests of maximal anaerobic power on a cycle ergometer were proposed [6–11]. The differences between these protocols of all-out cycling exercise mainly concerned the value of the load (i.e. the braking force) or the duration of exercise.

However, the validity of the results of these jump, staircase, or cycling tests was questioned. Indeed, well known experimental studies on mechanical properties of isolated muscles that were performed between 1935 and 1940 [12–14] have found that (1) the force production depends on the speed of shortening; (2) the force-velocity relationship can be described with an exponential [12] or hyperbolic equation [13]; (3) the parameters of these relationships (maximal isometric force, maximal velocity, curvature of the relationship) largely depend on the types of muscle fibers; (4) maximal power (*P*_max⁡_) corresponds to optimal values of force (*F*_opt_) and velocity (*V*_opt_); (5)  *P*_max⁡_, *V*_opt_, and *F*_opt_ largely depend on muscle fiber types. The results of these first experiments carried out in frog muscles at low temperatures were confirmed by more recent studies in mammalian muscles at physiological temperatures [15, 16], in human fibers [17], and in mammalian or human skinned muscle fibers [18, 19]. The main results of these studies are developed in Appendix A.

In vivo, a hyperbolic force-velocity relationship during maximal voluntary contractions against different load was first observed in amputees [20]. Thereafter, the same hyperbolic relationship was observed for maximal voluntary contractions during monoarticular exercises such as elbow flexion, provided that the inertia and acceleration of the forearm were taken into account in the computation of the actual force exerted by the muscles [21]. In rehabilitation, isokinetic ergometers were soon used in the study of the relationship between force (or torque) and angular velocity, especially for the knee extensor and flexors [22].

Because of the dependence of *P*_max⁡_, *V*_opt_, and *F*_opt_ on muscle fiber types, it is difficult to know the optimal conditions (loads or velocities) which correspond to the production of *P*_max⁡_ before the completion of the above mentioned cycling tests. As a consequence, different protocols have been proposed for the measure of *P*_max⁡_ of the legs or the arms and for the determination of force-velocity relation in cycling on friction-braked ergometers [23] or isokinetic cycle ergometers [24]. Currently, there is no consensus on the optimal protocol for the estimation of *P*_max⁡_ from torque-velocity (or force-velocity) relationships or single all-out cycling exercises. Although the validity of the Wingate test is debated, it is likely that this anaerobic test is the all-out cycling test which is currently the most often used not only in athlete testing but also in studies on the biological adaptation to strenuous exercise. For example, more than 600 articles are listed when the data bank PubMed is questioned about the Wingate test.

The effects of training, athletic practice, diet, and pharmacological substances upon the production of maximal mechanical power will not be discussed in this paper mainly focused on the methodology and limiting factors of all-out tests and *P*_max⁡_ in cycling. The influence of biochemical factors (acidosis and alkalosis, phosphate ions, etc.) upon the results of the all-out tests probably depends on the protocol. Therefore, the bioenergetics of the all-out cycling exercises is recalled in the present review in addition to the biomechanical characteristics specific of maximal and high speed cycling. Thereafter, the different protocols are presented before the discussion of the technical and biological factors that determine the results to these tests.

## 2. Biomechanics of High Speed and High Power Cycling 

The biomechanics of submaximal cycling is presented in Appendix B. The following lines present some particularities of high speed versus low speed cycling and maximal versus submaximal cycling exercises.

**Figure 1:**
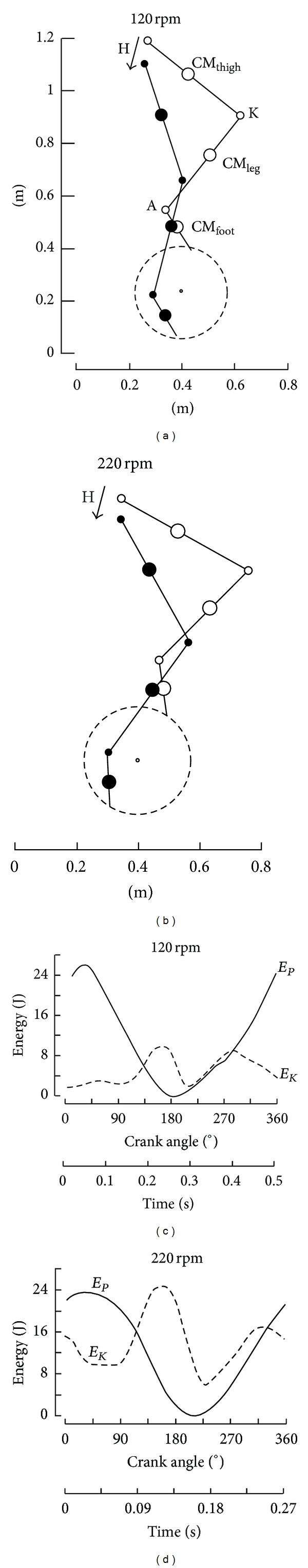
(a) and (b) modelling of cyclist legs with three rigid segments; H, K, A correspond to hip, knee, and ankle joints; black dots and empty circles correspond to the centers of mass of the thighs, lower legs, and feet; dotted circles correspond to pedal trajectory. (c) and (d) changes in gravitational potential energy *E*_*P*_ (continuous lines) and translational kinetic energy *E*_*K*_ (dotted line). Comparison of experimental data collected in a track cyclist on a Lode ergometer at 120 rpm ((a) and (c)) and 220 rpm ((b) and (d)). Personal data.

**Figure 2:**
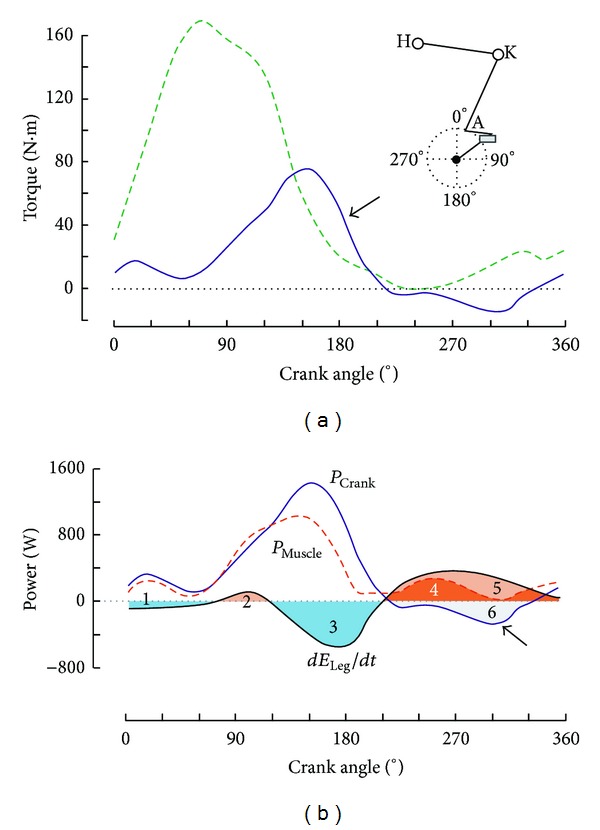
(a) Comparison of the torques exerted on the crank at 90 (green dashed line) and 180 rpm (blue line). (b) Data at 180 rpm: *P*_Crank_ (blue line) power exerted on the right crank; *dE*_Leg_/*dt* (black line) variations in mechanical energy of the right leg; *P*_Muscle_ power (red dashed line) output by the leg muscle equal to the difference between *P*_Crank_ and *dE*_Leg_/*dt*. Area 4, muscle contribution to the increase in *dE*_Leg_/*dt* during upstroke. At constant crank velocity, area 1 + 3 + 6 is equal to area 2 + 4 + 5. Personal data.

With the other things being equal (same pattern of angular movements at the ankle, knee, and hip), the variation in potential energy (Δ*E*_potential  leg_ expressed in joules) within a pedal revolution is independent of pedal rate. But, the rate of variation in potential energy (*dE*_potential  leg_/*dt* expressed in watts) is proportional to pedal rate in this case (Figures 1(c) and 1(d)). Kinetic energy is function of the square of velocity, and its importance largely increases with pedal rate (Figures 1(c) and 1(d)). Consequently, a larger transformation of the kinetic energy of the legs into mechanical work at the crank level is a possible explanation of the shift of the peak torque and the higher torque at the end of downstroke at high pedal frequency. Peak torque during a revolution is observed around 90° at low and medium pedal rates [25]. But at the peak velocity (≥200 rpm) of an all-out test against the inertia of the flywheel, peak torque occurs at pedal angles between 140 and 150° (Figure 2), that is, before the end of the downward pedal motion [26, 27]. As previously suggested in studies on submaximal cycling at 90 rpm [28] or between 60 and 120 rpm [29], most of the decrease of the segmental energy benefits the power transfer to the pedal. The clear opposition of *P*_crank_ and *dE*_Leg_/*dt* observed during downstroke at high pedal rates (Figure 2(b)) is in agreement with this hypothesis of an energy transfer even at high pedal rate. Cycling is a movement with several degrees of freedom, and the higher torque at the end of the downwards pedal motion can also be partly explained by differences in leg segment positions at low and very high velocities (Figures 1(a) and 1(b)).

In maximal sprint cycling, the use of the inverse dynamic technique has shown that most of the power during downstroke is produced at the hip instead of the knee as in submaximal cycling and that hip extension power is twice as great as knee extension power [30, 31]. These results do not mean that most of the mechanical work is performed by the hip extensor muscles instead of the knee extensor muscles during maximal cycling. Indeed, the coactivation of monoarticular knee extensors (the quadriceps muscles) and biarticular hip extensor-knee flexor muscles (the hamstrings) enables the energy transfer between hip and knee joints (see Appendix B). The first electromyographic studies on maximal cycling have found an increase in the contribution of knee flexors during upstroke at high velocity cycling (>200 rpm) [32] or at the end of an all-out 45-second exercise [33]. While submaximal cycling exercises are mainly performed with a reliance on knee extension and small contributions from knee and hip flexions, there was an important positive contribution from the muscles acting during the upstroke phase (almost 14% of maximal power output on the entire cycle) in a study on maximal sprint on a cycle ergometer [34], which confirmed the results of a simulation study [35]. During submaximal cycling at low pedal rate, the subjects generally do not pull on the pedal and a negative torque is observed during upstroke [36]. In contrast, during maximal exercise with toe clips, the subjects pull on the crank and the measured torque is positive during the whole pedal revolution at low and medium velocities. The study of joint-specific powers by the inverse dynamic method indicates that the contribution of knee flexion power over a whole revolution is approximately equal to the contribution of knee extension power during a maximal exercise [30, 31]. Consequently, in all-out cycling, a forth functional group (the uniarticular hip and knee flexors or FLEX, see Appendix B) should be added to the 3 synergies proposed by Hug et al. [37] for submaximal cycling. However, a negative torque is observed even with toe clips during upstroke at very high velocity (Figure 2(a)) [26, 27], which indicates a pedal contribution to the increase of leg mechanical energy between 180 and 360°. The contribution of flexor muscle activity during upstroke can be computed by substracting *dE*_Leg_/*dt* (computed from video data) from *P*_crank_ [38, 39]. In spite of a negative crank torque during upstroke at very high pedal rate, the muscular contribution to leg flexion is not negligible (area 4 in Figure 2(b)). When compared with submaximal exercises, the contributions of knee and hip flexion to power output increase during all-out cycling with toe clips and straps, and it is likely that all the muscle groups of the leg contribute to power production. However, it is possible that the activation levels of the gluteus maximus, hamstrings, tibialis anterior, and tensor fasciae latae are submaximal (<80%) during all-out cycling as suggested by the comparison with EMG activities during maximal voluntary contraction in isometric and isokinetic modes [40]. Plantar flexors should be able to produce high force levels at high shortening velocities in order to contract concentrically during knee extension and produce power. Moreover, high values of ankle torque are necessary for the transfer of the leg mechanical energy in addition to the work produced by the hip and knee extensor muscles. Consequently, it is possible that, at very high pedal rate, the ankle torque necessary to the leg-crank energy transfer becomes equal or higher than the torque corresponding to the maximal isometric contraction of the plantar flexor muscles at the end of the pedal downstroke. In a study on four cyclists, the contraction was eccentric for the biarticular ankle plantar flexors (gastrocnemii) in three subjects [39].

According to Freund [41], the rate limiting factor for alternating movements could be the subtraction of counteractive forces generated by the two antagonistic muscle groups: the contraction of the antagonistic muscle is superimposed on the relaxation of the agonist muscle. In cycling, the subtraction of counteractive forces could correspond to the actions of the contracting muscles of one leg and relaxation of the homologous muscles of the other legs: the muscles activated during the beginning of the downstroke of the left leg are the antagonist of the muscles activated during the beginning of the upstroke [42]. It is possible that the active muscles at the beginning of the upstroke have to offset the active state and an insufficient relaxation of active muscles during the downstroke [29, 35, 43]. This effect is assumed to be important at high movement frequencies and could limit not only maximal pedal rate but also optimal pedal rates (*V*_opt_) and maximal power output [44] in agreement with the result of an experimental study on mouse isolated muscle [45]. Moreover, the higher the pedal rate, the earlier the activations of the different muscles within a pedal revolution because of their electromechanical delays [40, 46].

In summary, the variations in leg mechanical energy within one revolution increase with pedal rate, which results in (1) a higher contribution of nonmuscle forces to the torque exerted on the crank; (2) a shift of peak torque production toward the end of the downstroke at high pedal rates. Therefore, it is the values of power or torque averaged over one revolution that must be used in the assessment of maximal power output by the leg muscles or in the determination of the relationships between force (or torque) and velocity (or pedal rate). 

## 3. Bioenergetics of Short All-Out Exercises

Paradoxically, the first protocols of maximal power assessment were not proposed to determine the mechanical properties of the legs or the arms. Indeed, the prevailing models of athletic performances were mainly based on exercise bioenergetics not biomechanics. The purpose of the short all-out sprint protocols was the assessment of the maximal power of the anaerobic metabolism, that is, the maximal rate of anaerobic ATP synthesis. The maximal mechanical power was assumed to be the expression of the maximal rate of anaerobic ATP synthesis. The long-lasting all-out exercise protocols were designed for the assessment of the maximal anaerobic capacity, that is, the maximal amount of ATP which can be supplied by the anaerobic metabolism. The maximal amounts of work performed during these tests were assumed to be the expression of the maximal amount of ATP which can be supplied by the anaerobic metabolism.

It is likely that the ATP resynthesis during a single all-out exercise lasting less than 5 seconds is mainly provided by anaerobic alactacid metabolism [47–50], that is, the breakdown of creatine-phosphate in creatine + inorganic phosphates. The energy supply of maximal exercises shorter than five seconds was first considered to depend mainly on creatine-phosphate breakdown, and the performances in these tests were considered as the expression of maximal alactic power.

It is likely that, during an all-out exercise, creatine-phosphate breakdown is higher in fast muscle fibers compared to slow fibers. For example, in type IIA fibers, [PCr] decreased to 46.6% of resting values after a 10-second all-out cycling exercise at 120 rpm, whereas the change in [PCr] was 53.9% in type I fibers [51]. In the same time, [PCr] was reduced to about 39.0% of resting values in the fibers expressing both IIA and IIX myosin heavy chains.

Creatine-phosphate (*pK*_*a*_ = 4.5) is more acid than creatine (*pK*_*a*_ = 6.8), and its breakdown in creatine + inorganic phosphate corresponds to an uptake of n hydrogen ions [52], which depends on pH (*n* = 0.38 and 0.70 moles for muscle pH = 7 and 6.4, resp.). A transient muscle alkalinization has been observed at the beginning of electrically stimulated contraction of isolated muscles [53, 54]. In a simulation of an all-out running sprint, the first five seconds corresponded to a muscle alkalinization [55]. 

Inorganic phosphates correspond to monoprotonated and diprotonated phosphate ions whose proportions depend on pH:

(1) $$\begin{array}{c} {{\text{HPO}}_{4}}^{2-}+{\text{H}}^{+}⟷{{\text{H}}_{2}{\text{PO}}_{4}}^{-}\\ \text{monoprotonated}\;{\text{P}}_{\text{i}}+{\text{H}}^{+}⟷\text{diprotonated}\;{\text{P}}_{\text{i}}. \end{array}$$

A large proportion of the phosphate ions should correspond to monoprotonated phosphate at the very beginning of exercise because of muscle alkalinization. The muscle fatigue due to the accumulation of phosphate ions resulting from creatine-phosphate breakdown is mainly due to diprotonated ions [56, 57]. Therefore, it is possible that the fatigue due to the deleterious effect of phosphate accumulation upon force and shortening velocity is not important at the very beginning of an all-out exercise because of muscle alkalinization.

Muscle biopsies of the quadriceps muscle taken at the end of 10 all-out cycling exercises indicate that lactate production begins earlier than it was previously assumed [58]. This early lactate production is also suggested in the simulation of an all-out 100 m run: the rate of lactate production is high after 5-6 seconds [55]. This increasing production of lactic acid counterbalances the initial muscle alkalosis and pH return to a value close to its initial value around the 10th second in this model [55]. Beyond the 10th second of an all-out test, the glycolytic and aerobic metabolisms provide most of the ATP resynthesis because of the depletion of creatine-phosphate [59].

The lactate concentration at the 30th second of an all-out test was only twice the concentration observed at the 10th second [58]. This lactate concentration lower than expected at 30 seconds could be explained by (1) a decrease in ATP hydrolysis; (2) an inhibition of glycolytic enzymes by acidosis; (3) lactate efflux outside the muscle fibers; (4) an increasing contribution of the aerobic metabolism. The activities of glycogen phosphorylase and phosphofructokinase are inhibited by acidosis, and the glycolytic rate corresponding to pH at the end of a 30-second all-out test should be approximately 50% lower than at the beginning [47, 60]. There is a lactate efflux outside the muscle fibers during a 30-second all-out test. However, blood lactate at the end of this exercise is much lower than muscle lactate, and several minutes are necessary for equilibration between muscle and blood lactate [60–63]. This lactate efflux depends on capillary supply which is more developed around slow fibers [64] and is improved by training.

Aerobic metabolism has been estimated to provide 9–40% of the energy utilised during a 30-second all-out test in function of the age and training status of the subjects [62, 65, 66]. The aerobic contribution to power production increases with the duration of supramaximal exercises [67, 68], and maximal oxygen uptake is reached during all-out tests lasting from 60 to 90 seconds [69].

The duration of a 30-second all-out test is too short to solicit the maximal anaerobic capacity. Indeed, power output at the 30th second of an all-out test is higher than the power output corresponding to maximal oxygen uptake [70]. Therefore, the cumulated oxygen deficit during an all-out test should increase beyond 30 seconds. Similarly, a 30-second all-out test is too short for maximal accumulation of lactic acid [62, 67, 71]. In spite of the high concentration of muscle lactate (120 mmoles·kg dry weight-1), the value of pH (6.7) at the end of a 30 second all-out test measured by Bogdanis et al. [59] was less acidic than the values observed in some protocols of short exhausting exercises (pH from 6.26 to 6.57) according to Hultman and Sahlin [52]. In another study, the anaerobic ATP production (creatine-phosphate breakdown + anaerobic glycolysis) was 32% less for 30 s of exhausting exercise than for 2 min of exhausting exercise [68].

Several protocols designed for the assessment of *P*_max⁡_ consist in the repetitions of all-out exercises against different loads. The contribution of fast muscles fibers to power output is important during high-power exercises [51, 72–74]. In addition, the capillary network around fast fibers is less developed, which should limit lactate clearance [64, 73]. Therefore, the recovery of power production should be longer in fast fibers because of higher levels of ATP and phosphocreatine breakdown and lactate accumulation [51]. The occlusion of the circulation immediately after exercise impedes creatine-phosphate resynthesis and pH restoration [75, 76], which demonstrate the aerobic resynthesis of creatine phosphate and the importance of blood circulation. In the case of repeated sprints, the intervals between exercise bouts should be long enough for the recovery in the most powerful subjects who possessed higher percentages of fast muscle fibers but, generally, lower aerobic potential.

Muscle pH recovers slowly, and the inhibition of the glycogen phosphorylase and phosphofructokinase activity by acidosis slowly disappeared [47], and the proportion of diprotonated inorganic phosphate should stay high because of muscle acidosis. Therefore, it is not possible to repeat long-lasting all-out cycling exercises (30–45 s and more) in the same session. In contrast, creatine-phosphate returned to 65 and 85% its initial value at 90 seconds and 6 minutes of recovery after a 30-second all-out test.

The assessment of maximal power is often included in session when other physical tests are performed (direct or indirect assessment of maximal oxygen uptake…). The possibility to produce maximal power after a preliminary exercise depends on the intensity and duration of this previous exercise. The value of *P*_max⁡_ fully recovers one minute after the completion of a cycling exercise at submaximal rate (60–80% *V*_O2_max⁡) [77]. In contrast, maximal power output was only equal to 87% *P*_max⁡_, 8 minutes after an exercise at 120% V_O2_max⁡ [77].

In summary, creatine-phosphate breakdown supplies ATP during the first seconds of all-out exercises. At the very beginning of exercise (<5 s), the effect of diprotonated phosphate accumulation is probably limited by the muscle alkalinization due to phosphocreatine breakdown. ATP synthesis by the lactic metabolism increases from the first seconds of exercise, and its contribution to energy supply is important beyond 5 seconds. Therefore, muscle acidosis potentiates the deleterious effect of diprotonated phosphate accumulation. During long-lasting all-out sprint ATP hydrolysis progressively decreases and the contribution of the aerobic metabolism prevails. The duration of a 30–45-second all-out test is too short to solicit the maximal anaerobic capacity and maximal lactate accumulation. The recovery of creatine-phosphate stores is aerobic, and, in the case of repeated sprints, the intervals between exercise bouts should be long enough for the recovery in the most powerful subjects.

## 4. Expression of Optimal Braking Force and Power Output

Maximal power output (Appendix A) corresponds to optimal values of force (*F*_opt_) and velocity (*V*_opt_). The way in which *F*_opt_ is generally expressed in cycling exercises (for example 75 g·kg^−1^ body mass) is considered as incorrect [78]. Body mass (BM) of humans is reported in kilograms as mass is the amount of matter in a body, but grams do not correspond to a force. Braking force should be expressed in newtons and body weight (BW); that is, the force exerted by gravitational attraction on body mass should also be reported in newtons (BW = 9.81 BM). However, the ratios kg·kg^−1^ BM (or g·kg^−1^ BM) and N·N^−1^ BW are dimensionless. In the present paper, optimal force is expressed as a percentage of body weight (for example 7.5% BW) [78].

Optimal braking force in cycling should depend on the strength of the subject [79] and be proportional to the cross-sectional area, that is, BM^0.66^. Therefore, in theory, *F*_opt_ should be equal to *X* ^0.66^% BW. Consequently, with other things being equal, *F*_opt_ should be higher in small subjects, which is not the case in children. When expressed as a percentage of body weight, *F*_opt_ should be excessive in overweight people. There was no significant difference between obese and nonobese adolescents when *F*_opt_ was related to lean body mass, whatever the use of standard or power function ratios [80].

Nonetheless, the force exerted on the flywheel has no biological meaning because it depends not only on the force exerted on the pedal but also on the design of the cycle ergometer. The work performed during one pedal revolution against a braking force *F* is equal to the product of *F* and the meters of development (*D*), that is, the distance travelled by a point of the rim for each pedal revolution. Nowadays, the values of *D* of the friction-braked ergometers are generally equal to 6.11 m, which facilitates the calculation of power (*P*  in watts = load in kilograms × pedal rate in rpm). In the past, *F*_opt_ was sometimes expressed as J·kg^−1^ BM because of differences in the value of *D* between the available cycle ergometers. For *D* = 6.11 m, the work corresponding to one revolution against the optimal force 7.5% BW is equal to 4.5 J·kg^−1^ BM (0.458 kgm·kg^−1^ BM), which corresponds to an optimal force equal to 4.58% BW for another ergometer with a value of *D* equal to 10 m.

Muscular power output is proportional to the muscle cross-sectional area and fiber length and, consequently, proportional to the muscle volume. Therefore, *P*_max⁡_ should be related to active muscle volume (*P*_max⁡_·L^−1^ active muscles) when the study is focused on the assessment of the contractile properties of the skeletal muscles. Indeed, in isolated muscle, *P*_max⁡  muscle_ related to muscle volume largely depends on muscle fiber types [16, 18, 19]. Moreover, *P*_max⁡_·L^−1^ should be independent of the body dimensions, arm levers, and pennation [81]. With the other things being equal, it is implicitly assumed that the active muscle volume is proportional to the leg muscle volume for all-out cycling exercises (or arm muscle volume for all-out cranking exercises). *P*_max⁡_ can be related to muscle volume determined from the sum of incremental volumes (equal to the products of slice thickness and cross-sectional area) obtained with magnetic resonance imaging [82]. However, this method is time consuming and expansive. Therefore, *P*_max⁡_ is generally related to some indirect indices of muscle volume such as thigh muscle area estimated from tomodensitometric radiographs [83], leg volume (lean leg volume or lean thigh volume) estimated by means of anthropometric techniques [84, 85], or quadriceps volume [86], estimated by means of a regression equation derived from autopsy studies [87]. Maximal power output can also be related to lean body mass [80]. However, the measure of lean body mass is difficult with the usual methods (skinfold) in obese subjects and should be determined by dual-energy X-ray absorptiometry (DEXA) [80]. Finally, the values of the different power indices (*P*_max⁡_, peak power, or PP_corr_) are generally also related to body mass (*P*_max⁡_·kg^−1^ BM) in nonobese subjects because it is the easiest way to take into account anthropometric differences between subjects. Moreover, it is generally the only variable which can be compared between studies that use different methods for the estimation of muscle volume. As *P*_max⁡_·L^−1^, the value of *P*_max⁡_·kg^−1^ BM is considered as an expression of the contractile properties of the active muscle mass in nonobese subjects (see chapter on *P*_max⁡_ and muscle fibers).

However, it has been suggested that the use of such ratios to construct standards could be fallacious and misleading, and it has been proposed to use regression standards that describe the relationship between variables [88]. However, the expression of *P*_max⁡_ must be adjusted to the aims of its determination. In some cases, the use of regression between variables is probably the best use of *P*_max⁡_ when the purpose is to construct standards, provided that the data are collected in large populations. For example, an allometric scaling of Wingate test performances for body mass and lean body mass was studied in college women [89] or in children and adolescent [90] or young basketball players [91] with inclusion of gender and age in the models. 

But, in many other cases, the expression of *P*_max⁡_ must be adjusted to the biomechanical constraints of the physical activity. The value of *P*_max⁡_ should be expressed in absolute value (W) when power production without any restriction in the body mass of the subject is the main factor limiting performance. The assessment of *P*_max⁡_ during a cranking exercise in the grinders of the America's cup is a good example of such an expression of power output [92]; the absolute value of *P*_max⁡_ in grinders (1420 W in cranking) was the main information in this paper. The value of *P*_max⁡_ should be related to body mass of the subject (*P*_max⁡_·kg^−1^ BM) when short accelerations of the body mass are factors limiting performance as for example in sprint, track cycling, soccer, handball, volleyball, and so forth. In theory, *P*_max⁡_ should be related to body weight (instead of body mass), when short exercises against the gravitational force are factors limiting performance (soccer, handball, volleyball, etc.). However, the variations in gravitational force can be considered as negligible on earth and there is no need to relate *P*_max⁡_ to BW in addition to BM. *P*_max⁡_ should be related to body surface when aerodynamic resistance is a limiting factor, for example, maximal speed in track cycling. 

## 5. The Wingate Anaerobic Test 

The Wingate anaerobic test (generally called “Wingate test”) first presented by Ayalon et al. [93] was derived from the test previously proposed by Cumming [94]. Thereafter, Bar-Or [6, 7] published comprehensive studies of the Wingate test and its applications. The Wingate test consists in pedalling with maximal (all-out) effort for 30 seconds against a constant braking force (7.5% BW for a Monark ergometer). Mean pedal rate is measured for each 5-second interval. For the Monark ergometers, mean power outputs corresponding to these intervals are given by the product of braking force and mean pedal rate. 

Three indices of anaerobic performance are computed: peak power output (PP), mean power output (MP) over the 30 seconds of the whole test, and the decrease in power (fatigue index). In the first description of the test, peak power output corresponded to the highest 5-second mean power and the fatigue index was calculated as the difference between peak power output and the lowest power output of the successive 5-second intervals. Nowadays it is easy to measure the pedal rate at a high sampling frequency, and peak power is generally measured more accurately over a shorter time than five seconds (for example each second or over one revolution). Before the test, the subjects pedal at low pedal rate with a low resistance for a few minutes. This warm-up exercise is generally interspersed with two or three all-out sprints lasting only two to three seconds. Then, the subjects rest on the ergometer before the start. With the cycle ergometers available between 1970 and 1980, it was difficult to set the braking force before the subjects began to pedal. Therefore, the Wingate test started from a rolling start, around 60 rpm, against a low resistance, and then the load was rapidly set.

Other durations of all-out cycling tests were proposed such as a 40-second all-out test against a constant load equal to 5.5 kg [8, 95]. Detrimental physical responses (dizziness, headaches, nausea, vomiting, etc.) and subsequent subject apprehension have been reported to occur after the Wingate test. The mean power output during the 30 seconds of a Wingate test was highly correlated with the mean power measured during the first 20 seconds of the same exercise [96], which was confirmed by a study comparing 20- and 30-second all-out tests performed during different sessions [97]. An exponential regression equation was proposed to predict the performance in a “normal” Wingate test from the data of a 20 second all-out test. Therefore, a 20-second all-out test could be proposed in the place of the 30 second Wingate test. Leg fatigue was the only detrimental side effect reported following a 20 second all-out test, which should improve the reliability of the protocol and the compliance to the test.

The fatigue index was the least reliable of the three Wingate test indices, and its validity was questioned as it largely depends on aerobic performance. Consequently, peak power and mean power output were the main topics of most studies. Nonetheless, the validity of mean power as an index of anaerobic capacity is as questionable as the validity of the fatigue index [67, 68, 71, 98–101]. The aerobic metabolism provides a higher contribution to this energy demand in endurance athlete than in sprint athletes [99]. Therefore, peak power during a Wingate test is probably the only index that merits to be measured, provided that the load is optimal. However, a 30-second all-out test is exhausting, and it is not possible to test the subject with another load after a long recovery. In two other studies, it has been proposed to repeat short sprints (5–7 seconds) against different loads on a Monark ergometer with 3–5-minute recovery intervals and to measure peak power, only [9, 10]. The highest value of peak power (product of peak pedal rate *V*_peak_ and loads) was considered as the maximal anaerobic power if *V*_peak_ rate corresponding to this trial was close to 110 rpm.

## 6. Force-Velocity Tests on Cycle Ergometers

A protocol of all-out cranking exercise was designed to estimate the strength and speed characteristics in addition to the only assessment of *P*_max⁡_ [102]. A curvilinear relationship was expected as observed in mammalian isolated muscles or in monoarticular exercises in humans (see Appendix A). Therefore, the computation of the curvature indices (*a*/*F*_0_) was planned to suppress the effect of body dimensions, arm levers, and muscle pennation angles on the values of *V*_0_ and *F*_0_ [103]. This test derived from the protocol proposed by Pirnay and Crielaard [10] consisted in measuring peak pedal rate (*V*_peak_) on a Monark cycle ergometer with handles in place of pedals, during short maximal all-out cranking exercises (about 6 s) against many different braking forces (*F*). Indeed, a large number of experimental force-velocity data is generally necessary to compute curvature indices. 

**Figure 3:**
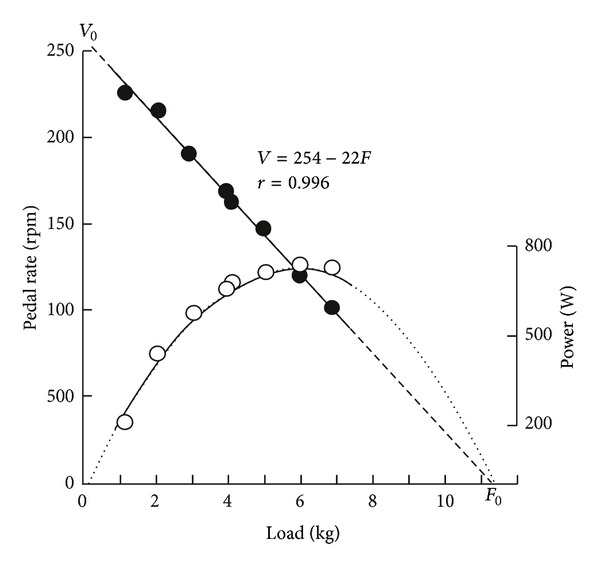
Black dot, relationship between peak pedal rate during all-out cranking exercises against different loads on a Monark cycle ergometer; empty circle power output at peak pedal rate. Data collected on a hand-ball player, adapted from Vandewalle et al. [23], with permission.

**Figure 4:**
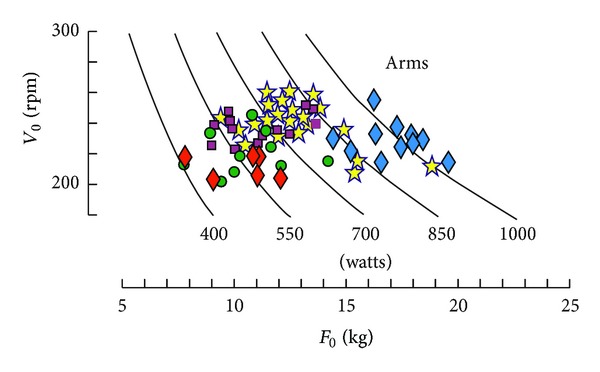
Parameters *V*_0_ and *F*_0_ of the individual force-velocity relationships on a Monark cycle ergometer; yellow stars, male boxers; green circle, male recreational athletes; squares, male recreational tennis players; blue and red diamonds, male and female canoeists and kayakists who prepared the 22th Moscow Olympic game. Adapted from Vandewalle et al. [23], with permission.

The force-velocity relationship in cranking (Figure 3) was first studied for cranking exercise in elite subjects practicing canoeing, kayaking, hand-ball, and boxing (Figure 4). The test began with a load equal to 1 kg. After 5 min of recovery, the braking force was increased by 1 kg, and the same exercise was performed again until the subjects were unable to reach a peak velocity higher than 100 rpm. The relationship between peak velocity and braking force was computed according to the least square method. The first and second bouts (1 and 2 kg) were considered as warming-up and learning exercises and were performed again at the end of the test. Therefore, the subjects generally performed 8 to 10 short all-out sprints, but the only second trials with 1 and 2 kg were taken into account in the computation of the force-velocity relationship. The linear relationship between *V*_peak_ and *F* computed according to the least square method was transformed:

(2) $$\begin{array}{c} V_{\text{peak}}=a-bF,\\ V_{\text{peak}}=\;V_{0}\left(1-\frac{F}{F_{0}}\right),\\ F=F_{0}\left(1-\frac{V_{\text{peak}}}{V_{0}}\;\right), \end{array}$$

with *V*_0_ and *F*_0_ equal to the intercepts with the velocity axis and force axis, respectively (*V*_0_ = *a* and *F*_0_ = *a*/*b*). Since a linear relationship between *F* and *V*_peak_ was observed, *P*_max⁡_ corresponded to an optimal pedal rate and an optimal load equal to 0.5*V*_0_ and 0.5*F*_0_, respectively. Consequently, *P*_max⁡_ was calculated as equal to

(3) $$\begin{array}{c} P_{\max}=0.5V_{0}*0.5F_{0}=0.25V_{0}F_{0}. \end{array}$$

Therefore, the individual performances could be presented on a *V*_0_-*F*_0_ plot where all the subjects with the same *P*_max⁡_ are located on the same branch of hyperbola (*V*_0_ = 4*P*_max⁡_/*F*_0_; Figure 4).

Some years later, a new model of Monark ergometer was available (Monark 864 with basket). This Monark ergometer enabled the use of higher braking forces and their setting before cycling. Therefore, the force-velocity test could be applied to leg exercises with some changes in the protocols [104, 105]. Indeed, it was not necessary to use a large number of loads to determine the force-velocity relationship because the observed relationship for cycling exercises was linear as it was previously observed for cranking exercises. Therefore, the numbers of exercise bouts was lower: 5 to 7 repetitions (4-5 different loads, with repetition of the first and second loads which were considered as warming-up and learning exercises). In male adults, the first load was 2 kg, and the increment was 2 kg instead of 1 kg for the arm protocol. The recovery interval was 5 minutes as in cranking force-velocity test. As for cranking exercise, the values of *V*_0_ and *F*_0_ were determined from the linear relationship between *F* and *V*_peak_. *P*_max⁡_ was computed as equal to 0.25*V*_0_ *F*_0_. The highest values in *P*_max⁡_ (>20 W·kg BM^−1^) and *V*_0_ (>260 rpm) were observed in elite athletes practicing sprint events in running or cycling, whereas *P*_max⁡_ was lower than 10 W·kg BM^−1^ in children and elite long distance runners [105]. Similar linear regressions were reported for the relationships between load and peak velocity [106] or between load and 5-second average velocity [107]. The force-velocity test was considered as a test of maximal alactic power until a significant contribution of anaerobic glycolysis was found even after the first load [108].

Interestingly, a linear relationship between pedal rate and braking force on a friction-braked cycle ergometer has previously been observed in 1928 [109]. However, Dickinson did not published this article to present a test of maximal power in human but to verify Hill's hypothesis that *“the average external force exerted during a muscular movement, carried out with maximal effort, may be regarded as equal to a constant theoretical force diminished by an amount proportional to the speed of movement”* (see Appendix C). The force-velocity relationship obtained with Martin's cycle ergometer was comparable with today's results (Appendix C). But, ten years later, Hill [13] proposed his famous hyperbolic (instead of linear) force-velocity relationship that was not based on internal frictional resistance in the muscles. The results of Dickinson [109] were forgotten by most of the muscle physiologists and, consequently, ignored by the people interested in physical testing.

## 7. Torque-Velocity Test on an Isokinetic Ergometer

In 1981, Sargeant et al. [24] proposed to determine the relationship between pedal rate and the torque exerted on the cranks of an isokinetic cycle ergometer, that is, an ergometer whose pedal rate was constant and maintained whatever the force exerted on the pedals. This device consisted in a bicycle ergometer modified by the addition of a 3 hp (around 2200 W) electric motor which drove the cranks through a variable-speed gear box. This bicycle ergometer enabled pedal rate to be set and maintained in the range 23–180 rpm. Torque was measured by means of strain gages bonded on the cranks (0.17 cm cranks). The relationship between crank angular velocity and torque averaged over one revolution was linear (*r* > 0.97) for the five subjects who participated in the study. When torques *T* were related to upper leg volume (N·m·L^−1^), the regression (average of the five subjects) between torque *T* and pedal rate *V* was

(4) $$\begin{array}{c} T=45.9-0.208V\;\left(r=0.979\right),\\ V=220-4.81T, \end{array}$$

which corresponded to *V*_0_ = 23.0 rad·s^−1^, *T*_0_ = 45.9 N·m·L^−1^, that is, about 3 N·m·kg^−1^ BM. A linear torque-pedal rate relationship was also observed in a study that used the same concept of cycle ergometer with pedal rate between 60 and 160 rpm [110, 111]. Pedal rates from 13 to 166 rpm could be used with this ergometer. However, testing was restricted to pedal rates above 50 rpm in the powerful subjects to avoid measurement errors due to the deformation of the cranks below 40 rpm. Lower pedal rates were used in women (i.e. less powerful subjects), and an exponential torque pedal rate relationship was observed between 11 and 160 rpm, in this study.

The relation between isokinetic pedal velocity and torque has also been studied on a cycle ergometer that controls the velocity and measures the tension of the chain (Fitrocycle, Fitronics, Bratislava) [112]. A linear relation between pedal rate and chain tension (average values of 60 subjects) has been found for pedal rate ranging between 50 and 140 rpm with 10 rpm increments:

(5) $$\begin{array}{c} F=-0.0574X+13.68\;\left(r=0.9962\right). \end{array}$$

The values of *V*_0_, *T*_0_, *P*_max⁡_ and the regression between *T* and *V* can be estimated from the data presented in this study:

(6) $$\begin{array}{c} V_{0}=236\;\text{rpm}=24.7\;\text{rad}\cdot {\text{s}}^{-1},\\ P_{\max}=15.3\;\text{W}\cdot {\text{kg}}^{-1}\text{BM},\\ T_{0}=2.48\;\text{N}\cdot \text{m}\cdot {\text{kg}}^{-1}\text{BM}. \end{array}$$

## 8. Corrected Peak Power [113]

The force exerted on the pedal is used not only for the rotation of the flywheel against the braking force *F* but also for the acceleration of the flywheel up to peak velocity. At peak velocity (*V*_peak_) flywheel acceleration is equal to zero, and the force exerted on the pedal is used for the rotation against the resistance *F*, only. Therefore, Lakomy [113, 114] and Bassett [115] proposed to calculate the force necessary for flywheel acceleration to transform this force in an equivalent load (*F*_acc_) and to add *F*_acc_ and *F* (*F*_corr_ = *F*_acc_ + *F*). Power output *P*_rev_ during each revolution is equal to the product of the velocity during this revolution (*V*_rev_) and *F*_corr_ (*P*_rev_ = *V*_rev_ *F*_corr_). According to the relationship between force and velocity, *F*_corr_ decreases while *V*_rev_ increases up to peak pedal rate. Corrected peak power (PP_corr_) corresponds to the maximal value of *P*_rev_ during the acceleration phase.

Lakomy calibrated his ergometer by determining the relationship between flywheel deceleration and load. The flywheel was set in motion at a speed equivalent to 150 rpm and the deceleration resulting from the load in the absence of pedalling. The deceleration curves were obtained from 105 to 0 rpm. Then a linear regression between deceleration and load was obtained, and this equation was transformed to compute *F*_acc_ during the all-out sprint from the measure of acceleration:

(7) $$\begin{array}{c} \text{Deceleration}\left(\text{rpm}/\text{s}\right)=18.1\times \text{load}+4.10,\\ F_{\text{acc}}=\frac{\left[\text{Acceleration}\left(\text{rpm}/\text{s}\right)-4.10\right]}{18.1}. \end{array}$$

If there was no fatigue during a short all-out sprint, PP_corr_ should be independent of the load *F* and should be equal to *P*_max⁡_:

- if the load is equal to *F*_opt_, *V*_peak_ is equal to *V*_opt_ and PP_corr_ = *V*_opt_ *F*_opt_ = *P*_max⁡_;
- if the load is lower than *F*_opt_, peak velocity is higher than *V*_opt_ and PP_corr_  corresponds to the highest value of *P*_rev_ during the acceleration phase, which correspond to the revolution when *V*_rev_ and *F*_corr_ are equal to *V*_opt_ and *F*_opt_, respectively;
- if the load is higher than *F*_opt_, *V*_peak_ is lower than *V*_opt_ and PP_corr_ is lower than *P*_max⁡_.

However, PP_corr_ was not independent of *F* [113]: PP_corr_ decreases (about 10%) with the increase in *F* from 5.5 to 11.5% BW. This result could be explained by fatigue because the values of *V*_opt_ are obtained later with high values of *F* (see chapter on fatigue). In this study, PP_corr_ also depends on sampling time (0.5 or 1 s), and it would be better to measure velocity averaged on a revolution instead of averaged over a given time.

The values of *P*_corr_ were compared with the values of *P*_max⁡_ computed from a force-velocity relationship determined with 4 loads in two studies [116, 117]. The correlations between PP_corr_ and *P*_max⁡_ were significant, but PP_corr_ was approximately 10% higher than *P*_max⁡_ in both studies. The lower value of *P*_max⁡_ compared to PP_corr_ could possibly be explained by an early fatigue effect because the force-velocity test corresponds to peak velocity instead of data collected during the acceleration phase.

On the other hand, the reliability of PP_corr_ was lower than that of *P*_max⁡_ [117]. The reliability of PP_corr_ could be improved by more accurate measure of acceleration and the repetition of the test in the same session. Moreover, it is now possible to determine power output during an all-out sprint by measuring directly the torques exerted on the cranks (or the forces exerted on the pedals) instead of computing *F*_corr_ from *F*_acc_.

In summary, the value of PP_corr_ is approximately 10% higher than *P*_max⁡_ calculated from the data of a force-velocity test because *V*_opt_ is reached earlier during the acceleration phase instead of peak velocity. On the other hand, the reliability of PP_corr_ was lower than that of *P*_max⁡_.

## 9. *P*_max⁡_ and Torque-Velocity Relationship during a Single All-Out Sprint

The determination of a torque-velocity relationship during a single all-out sprint [116, 118] was directly derived from the study by Lakomy on the correction of peak power. First, the flywheel inertia was measured from the regression between flywheel deceleration and load (see the previous). The relationship between crank torque (*T*) and crank angular velocity (*ω*) was studied during the acceleration phase of short (<7 s) all-out sprints. The average crank angular velocity *ω* during each revolution was measured up to peak velocity. For each revolution, the average torque *T* exerted on the pedal was calculated as equal to the sum of *T*_acc_ (the average torque necessary for flywheel acceleration during each revolution) and *T*_*B*_ (the torque necessary for flywheel rotation against the braking force *F*) as in the study by Lakomy [113]. The acceleration of the flywheel was computed every 50 ms from the flywheel velocity data given by a disc with 360 slots fixed on the flywheel, passing in front of a photoelectric cell (669 impulses for each pedal revolution).

**Figure 5:**
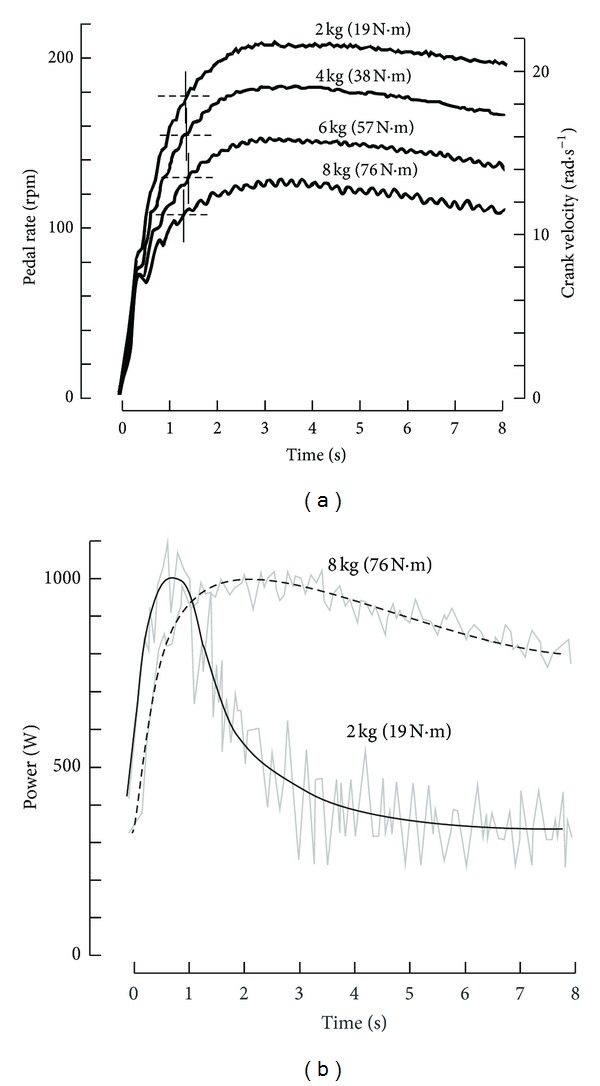
(a) Time pedal rate curve during all-out exercises performed by the same subject on a Monark ergometer against different loads (corresponding braking torque *T*_*B*_ in N·m in brackets), and crosses correspond to 85% peak velocity. (b) Time-power curves, in grey raw data, in black smoothed data. Adapted from Seck et al. [116], with permission.

**Figure 6:**
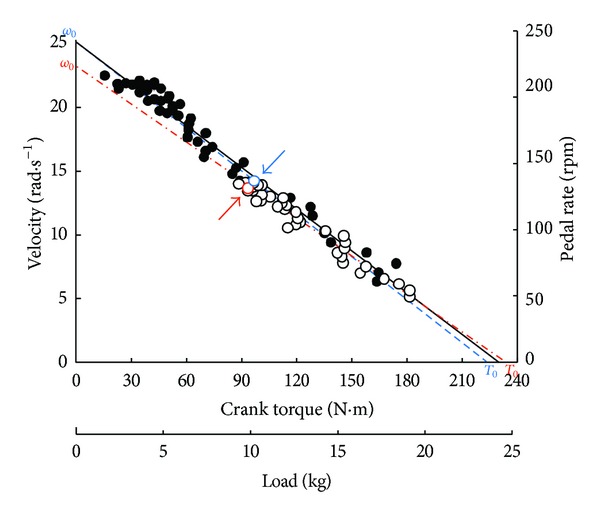
Relationships between crank torque *T* and crank angular velocity *ω* during all-out exercises on a Monark cycle ergometer against two braking forces *F*. Empty circles and red dashed line, data corresponding to *F* = 8 kg (*T*_*B*_ = 76 N·m); black dots and blue dashed line, data corresponding to *F* = 2 kg (*T*_*B*_ = 19 N·m); black line, *T*-*ω*  regression corresponding to all the data. Adapted from Seck et al. [116], with permission.

These all-out sprints were performed against 4 different braking forces (Figure 5) which corresponded to braking torques (*T*_*B*_) equal to 19, 38, 57, and 76 N·m at the crank level, that is, *F* equal to 2, 4, 6, and 8 kg. For each value of *F*, the individual relationships between *ω* and *T* could be described by a linear regression (Figure 6) and the values of *ω*_0_ and *T*_0_ for each load were determined by extrapolation from these individual regressions. The relationship between *V*_peak_ and *F* was also determined. The value of *P*_max⁡_ calculated from the usual *F*-*V*_peak_ relationship (*P*_max⁡_ = 0.25*V*_0_ · *F*_0_) was compared with *P*_max⁡ 2_ equal to 0.25*ω*_0_ *T*_0_ for each value of *F*. In addition, PP_corr_ was also calculated according to Lakomy (see the previous) for the different values of *F* [113, 114]. There was no significant difference between *P*_max⁡ 2_ and PP_corr_ that were 10% higher than *P*_max⁡_. The lower value of *P*_max⁡_ was interpreted as the effect of fatigue on *V*_peak_ that was reached later.

Similar linear *T*-*ω* relationships were obtained in another study [119]. This protocol has also been adjusted for the assessment of *P*_max⁡_ of the arms from a single all-out cranking exercise [120]. Thereafter, the computation of the torque-velocity relationship during a single all-out sprint according to this method was used to study the effect of fatigue induced by short exhausting or long-lasting exercises [121–124].

**Figure 7:**
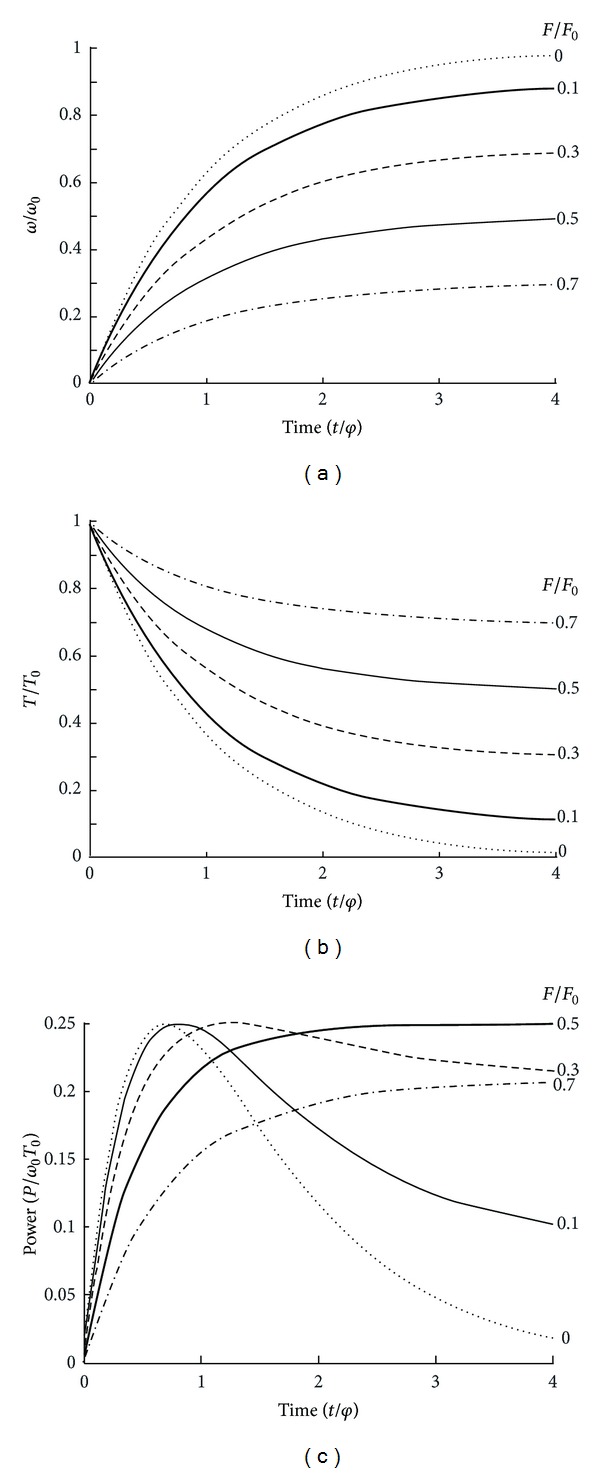
Theoretical time-crank velocity curve (a), time-torque curve (b), and time-power curve (c) against different braking forces *F* expressed as fraction of *F*_0_; pure inertial all-out exercises correspond to *F* = 0; time is related to time constant (*φ*).

It is can be demonstrated that, in the case of a linear regression (Figure 6) between pedal rate *V* and the maximal crank torque *T* corresponding to *V*, the relationship between *V* and time *t* is (Figure 7)

(8) $$\begin{array}{c} V=V_{0}\left(1-\frac{F}{F_{0}}\right)\left[1-e^{-t/\varphi }\right],\\ V=V_{\text{Peak}}\left[1-e^{-t/\varphi }\right], \end{array}$$

where *φ* is a time constant equal to

(9) $$\begin{array}{c} \varphi =\frac{2\pi \gamma v_{0}I}{9.81F_{0}r}, \end{array}$$

where *γ* is the gear ratio (for a Monark ergometer, *γ* = 52/14), *r* the radius of the flywheel, *I* the moment of inertia of the flywheel, *F*_0_ expressed in kilograms, and *v*_0_ = *V*_0_/60. The kinetics of *F*_corr_ and *P* during an all-out exercise (Figure 7) are

(10) Fcorr=F0(1−VV0)=F0−(F0V0)V,Fcorr=[FF0+(1−FF0)e−t/φ]F0,P=VFcorr,P  =  [FF0+(1−FF0)e−t/φ] ×[(1−FF0)(1−  e−t/φ)]F0V0.

If there were no fatigue and, consequently, no decrease in *V*_0_ and *F*_0_, the value of *V*_peak_ would be equal to the asymptote of this exponential model. The time constant of the curve (*φ*) is independent of load *F*. Therefore, the time necessary to reach a given fraction of *V*_peak_ corresponding to *F* is independent of *F* (Figure 5(a)). When braking force is low (black dots in Figure 6), the average pedal rate during the first revolution and *V*_peak_ are high. On the other hand, with a heavy braking force (empty circles in Figure 6), the average pedal rate of the first revolution and *V*_peak_ are low. In the case of an all-out sprint against a pure inertial load (*F* = 0), *V* at time *t* is given by the following equation: *V* = *V*_0_[1 − *e* ^−*t*/*φ*^].

If there was no fatigue during long-lasting all-out cycling exercises, the ideal solution would be a pure inertial load (*F* = 0) and a large increase in *φ* with the use of a cycle ergometer whose gear ratio *γ* and flywheel inertia *I* are high. Indeed, the torque-velocity relationship would be determined from a large range of *T*-*ω* data with this cycle ergometer. The pedal rate of the first revolution would be low, and a high peak pedal rate would be reached after many revolutions. Unfortunately, the effects of fatigue limit the increase in *φ* and, consequently, the increases in *γ* and *I*.

The pure inertial load was experimented with the use of an intermediate gear drive which increased the gear ratio to 7.43 : 1 [125]. The crank torque average over one revolution (*T*) was linearly related to pedal rate (*V*) averaged over one revolution (*r* = 0.99; *P* < 0.001)

(11) $$\begin{array}{c} V=236-1.16T. \end{array}$$

The values of *V*_0_, *T*_0_, *P*_max⁡_ and the regression between *T* and *V* (average values of 13 subjects) can be calculated from the data presented in this study:

(12) $$\begin{array}{c} V_{0}=236\;\text{rpm}=24.7\;\text{rad}\cdot {\text{s}}^{-1},\\ T_{0}=203\;\text{N}\cdot \text{m}=2.5\;\text{N}\cdot \text{m}\cdot {\text{kg}}^{-1}\;\text{BM},\\ P_{\max}=0.25V_{0}T_{0}=1253\;\text{W}. \end{array}$$

Interestingly, these values of *V*_0_ and *T*_0_ were equal to those in the study on an isokinetic Fitrocycle ergometer (see the previous) [112]. The value of PP_corr_ calculated according to Lakomy [113, 114] was 1317 W (16.4 W·kg^−1^ BM), that is, 5.1% higher than *P*_max⁡_. The average pedal rate corresponding to PP_corr_ was equal to 122 rpm, that is, 3% higher than *V*_opt_ (*V*_0_/2). Similarly the regression between the peak value of torque (*T*_IP_) within each half-revolution and *V* were linearly related (*r* = 0.99; *P* < 0.001):

(13) $$\begin{array}{c} V=242-0.758T_{\text{IP}}. \end{array}$$

Different cycle ergometers enable the measurement of the torque during cycling exercises. Therefore, it is possible to measure directly the torque exerted on the crank during an all-out sprint instead of computing the torque necessary to accelerate the flywheel. The torque pedal rate relationship during a single all-out sprint has first been studied by means of an electronic Lode Excalibur Sport Cycle ergometer, model with strain gauges bonded on to the crank. This cycle ergometer was used in the linear mode, that is, with a braking torque (*T*_*B*_) proportional to pedal rate (*T*_*B*_ = *Vf*_*i*_ where *f*_*i*_ is a proportionality factor). The torque-pedal rate relationships were determined with two values of *f*_*i*_: the lowest value (*f*_*i*_ = 0.001 Nm·s·rad^−1^; black dots in Figure 7) and a factor related to body mass (*f*_*i*_ = 0.00225 Nm·s·rad^−1^·kg^−1^ BM). At the beginning of an all-out sprint performed on this ergometer (Figure 2), the torque exerted on the crank reached a peak around 90° as observed during submaximal exercises at low and medium pedal rates [25]. At very high pedal rates (≥180 rpm), corresponding to the end of the all-out sprints, peak torque occurred before the end of downstroke at pedal angles between 140 and 150° (Figure 2(a)) [26, 27]. Moreover, at high pedal rates, crank torque at the end of the downstroke is higher (arrow in Figure 2(a)) than the torque measured at the same crank angle at the beginning of the same all-out sprint, that is, at a low pedal rate.

**Figure 8:**
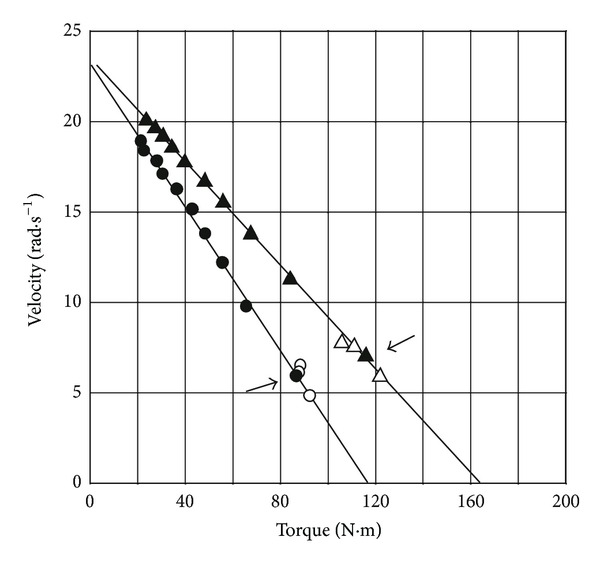
Relationship between crank torque (averaged over one revolution) and crank velocity during all-out exercises performed on a Lode ergometer in the linear mode for low (black symbols) and high (empty symbols) proportionality factors (*f*_*i*_) in two subjects.

The individual regressions between pedal rate (averaged over one revolution) and torque (averaged over one revolution) were linear (Figure 8) for all-out sprints performed not only with toe clips and straps [26] but also without toe clips [27]. 

The relation between force and pedal rate has also been studied on a cycle ergometer that measured the tension of the chain during an all-out sprint against a 20 N braking force exerted on the flywheel (Fitrocycle, Fitronics, Bratislava) [112]. The linear regression observed between pedal rate and torque was almost equal to the regression observed with the same ergometer in the isokinetic mode (see the previous):

(14) $$\begin{array}{c} F=-0.058X+13.58\;\left(r=0.9940\right). \end{array}$$

As for the isokinetic relationship obtained with the same ergometer in the same subjects (see the previous), *V*_0_, *T*_0_, *P*_max⁡_ (average values of 60 subjects) can be calculated:

(15) $$\begin{array}{c} V_{0}=236\;\text{rpm}\;=\;24.7\;\text{rad}\cdot {\text{s}}^{-1},\\ P_{\max}\;=\;14.8\;\text{W}\cdot {\text{kg}}^{-1}\;\text{BM},\\ T_{0}\;=\;2.39\;\text{N}\cdot \text{m}\cdot {\text{kg}}^{-1}\;\text{BM}. \end{array}$$

In summary, the parameters *V*_0_, *T*_0_ of the linear force-velocity (or torque-velocity) relationship and the value of *P*_max⁡_ can be assessed by means of a single short all-out sprint against the inertia of the flywheel. The values of the force (or torque) exerted at the crank level can be either computed from the acceleration of the flywheel or directly measured by strain gauges bonded on to the crank (or by a transducer measuring the tension of the chain). These single all-out sprints can be performed against a pure inertial load by increasing the flywheel inertia and/or the gear ratio. However, these single all-out sprints are often performed on usual cycle ergometers against the inertia of flywheel plus a small braking force.

## 10. Repeated-Sprint Cycling Test

Performances in many team sports (ice hockey, handball, soccer, etc.) depend on the ability to repeat short sprints [126], and, consequently, repeated-sprint cycling tests have been designed to mimic the activity on the field [127, 128]. In a protocol designed for soccer players, five short sprints (6 seconds) on a cycle ergometer are separated by 24-second recovery intervals, that is, one sprint every 30 s [127]. Total work done (*W*_tot_), total peak power (PP_tot_), work done during the first sprint 1 (*W*_1_), and peak power output during the first sprint (PP_1_) are recorded during this test. In addition, fatigue indices for work done (FI_*W*_) and peak power (FI_*P*_) are calculated from the decrement scores in work done (*W*_dec_) and peak power output (PP_dec_). In another protocol, the test consists of 4 repetitions of all-out cycling for 5 seconds against a resistance equal to 9.5% BW, separated by 10-second cycling at low intensity [128]. The topics of studies on the repeated-sprint ability (RSA) are the same as for the single sprint performance: reliability and validity of the results [127–129], metabolic and physiological limiting factors [130–133], effects of different training programs [134, 135], and chronobiology of performances [136, 137].

During single short-duration sprint, the contributions from creatine-phosphate breakdown and anaerobic glycolysis provide the majority of the total ATP production. Similarly, the depletion of creatine-phosphate stores is one of the limiting factors for the performance of repeated-sprint exercises as suggested by the strong relationship between the resynthesis of creatine-phosphate and the recovery of power output after a 30-second all-out sprint [49]. During repeated sprinting, the observed increase in aerobic metabolism probably explains the decrease in the relative contribution of anaerobic glycogenolysis [131, 132]. In contrast with single short-duration (<10 s) sprints, maximal oxygen uptake contributed to performance during repeated sprint (5 × 6-s max sprints every 30 s) [132]. Work decrement (%) was significantly correlated with muscle buffer capacity in addition to maximal oxygen uptake [133].

It is likely that the relative contributions of the different energy systems during repeated-sprint exercises depend on the exercise protocol (duration, number of repetitions, recovery duration, passive or active recovery) and training status [130]. There are few data regarding field-based, team-sport performance and the results of repeated-sprint tests. For example, the results of an experimental study suggested that the 5 × 6-s cycle test often used to assess RSA ability should be modified in function of sports [130]. Therefore, the movement patterns should be documented during competition before the choice of an RSA test specific of a team sport, which partly explain that there is no consensus on the protocols of repeated-sprint cycling tests.

## 11. Optimal Load of the Wingate Test

The question of the optimal force of the Wingate test has mainly been studied empirically by repeating this test with different loads in various populations. *A priori*, it was not obvious that the same load is optimal for the peak power, mean power, and fatigue index of the Wingate test. Therefore, another approach consisted in the comparison of the load optimal for the maximal power output in a force-velocity test (*F*_opt_ = 0.5*F*_0_) and the load optimal for peak power and mean power during all-out tests lasting 30 or 45 seconds [83, 104].

The braking force (7.5% BW for a Monark ergometers) maximising peak power output and/or mean power was first assessed in children. This optimal load was confirmed in a study using different loads (4, 6.5, 7.5, and 8% BW) in male or female children aged from 6 to 12 years [138]: there was no significant difference between 6.5, 7.5, and 8% BW but the performances were significantly lower with 4% BW. This optimal load equal to 7.5% BW (Monark ergometers) was later reconsidered in a study performed by the same research group at the Wingate Institute [139]. A new optimal value was proposed for adults (8.7% BW for Monark ergometers). Further studies indicated that this load (8.7% BW) was lower than the optimal load in nonobese male adults and that the optimal load was close to 10% BW [105, 140]. In another study the peak power with a load equal to 10% BW was on average 6% lower than the maximal power obtained from a force velocity test on the same ergometer [96]. Evans and Quinney proposed a regression which included body mass and leg volume to estimate optimal loads [141]. Higher peak power was obtained with the force predicted by this regression than with load proposed by the Wingate Institute [142]. However, Patton et al. who used the regression in a group of nonathletic military subjects, found that it had low validity [143].

The optimal load for Wingate mean power was determined from the data of a force-velocity test designed for the assessment of *P*_max⁡_  [83, 104]. This optimal load was not significantly different from the optimal load for *P*_max⁡_ (0.5*V*_0_) [83, 104]. Consequently, the same load should be optimal for both peak power and mean power during a 30-second Wingate test. 

The value of *P*_max⁡_ should be underestimated when the load proposed for average subjects is used in young, nonobese, powerful adults [144, 145]. However, in many cases, this underestimation is probably low because the relationships between power output *P* versus *F* or *V*_peak_ are quadratic:

(16) $$\begin{array}{c} F=F_{0}\left(1-\;\frac{V_{\text{peak}}}{V_{0}}\right),\\ V_{\text{peak}}=V_{0}\left(1-\frac{F}{F_{0}}\right),\\ P=V_{\text{peak}}F=V_{0}\left(F-\frac{F^{2}}{F_{0}}\right)=F_{0}\left(V_{\text{peak}}-\frac{{V_{\text{peak}}}^{2}}{V_{0}}\right). \end{array}$$

As *P*_max⁡_ = 0.25*V*_0_ *F*_0_, the relationships between power output *P* and *F* or *V*_peak_ are

(17) $$\begin{array}{c} P=4P_{\max}\left(\frac{V_{\text{peak}}}{V_{0}}-\frac{{V_{\text{peak}}}^{2}}{{V_{0}}^{2}}\right),\\ P=4P_{\max}\left(\frac{F}{F_{0}}-\frac{F^{2}}{{F_{0}}^{2}}\right). \end{array}$$

A value of *P* equal to 0.95*P*_max⁡_ corresponds to braking forces *F* equal to

(18) $$\begin{array}{c} 0.95P_{\max}=4P_{\max}\left(\frac{F}{F_{0}}-\frac{F^{2}}{{F_{0}}^{2}}\right),\\ \frac{4F}{F_{0}}-\frac{4F^{2}}{{F_{0}}^{2}}-0.95=0. \end{array}$$

With *f* = *F*/*F*_0_

(19) $$\begin{array}{c} f^{2}-f+0.237=0,\\ f=0.612\;\text{and}\;0.388. \end{array}$$

As *F*_opt_  corresponds to *f* = 0.50, *P*_peak_ equal to 0.95 *P*_max⁡_ corresponds to *F*_opt_ ± 22.4%. Similarly, the values of *V*_peak_ corresponding to 0.95 *P*_max⁡_ are equal to *V*_opt_ ± 22.4%. For example, the underestimation of *P*_max⁡_ is only 6.25% for *F* = 7.5% BW if the actual value of *F*_opt_ is equal to 10% BW. Therefore, an estimation of *P*_max⁡_ can be obtained with a simplified protocol (a Wingate test or short all-out sprints against 2 or 3 braking forces as proposed by Pirnay and Crielaard [10]. However, the value of *F*_opt_ is much higher in strong subjects and the underestimation could be larger [79]. In powerful subjects (*P*_max⁡_ higher than 15 W·kg^−1^ BM) [105], the underestimation of the maximal anaerobic power could be large because the braking force is much too low. The underestimation of *P*_max⁡_ would be 25% with a force equal to *F* = 7.5% BW in a subject whose *F*_opt_ is 15% BW. A large underestimation of *F*_opt_ probably explains the low value of peak power in a study on elite basketball players [145] where the braking force of the Wingate test was 7.5% BW.

## 12. Effects of the Material on *P*_max⁡_

### 12.1. Power Output at the Crank Level *Versus* Dissipated Power

The power output at the crank level is higher than the power dissipated at the flywheel level because of energy losses due to friction in the chain and sprockets. For the usual bicycles, this energy loss is often considered to be in the order of 5 to 9% [146]. For a Monark ergometer, the losses were about 2 and 4% for 150 and 300 W, respectively [147]. As the energy loss increased with power, it is likely that energy loss is around 10% beyond 1000 W. Therefore, the value of *P*_max⁡_ measured with strain gauge bonded to the cranks (or force transducer in the pedals) should be significantly higher than its value calculated from the energy dissipated at the flywheel level (work against braking torque + flywheel acceleration). These energy losses could partly explain the difference between *P*_max⁡_ measured with dynamometric crank on a Lode Excalibur ergometer and *P*_max⁡_ calculated from the results of a force-velocity test against different loads on a Monark ergometer in the same subjects [26].

### 12.2. Effects of Toe Clips and Crank Length

The use of toe clips improved all the performances (peak power, mean power and fatigue index) of a Wingate test performed with a load equal to 7.5% BW [142]. Similarly, in a study comparing the torque pedal rate relationships measured on a Lode cycle ergometer with and without toe clips, *P*_max⁡_ and *T*_0_ were significantly higher (+17 and +13%, resp.), but *ω*_0_  was unchanged. Moreover, the *T*-*ω* relationships were linear with and without toe clips [27].

**Figure 9:**
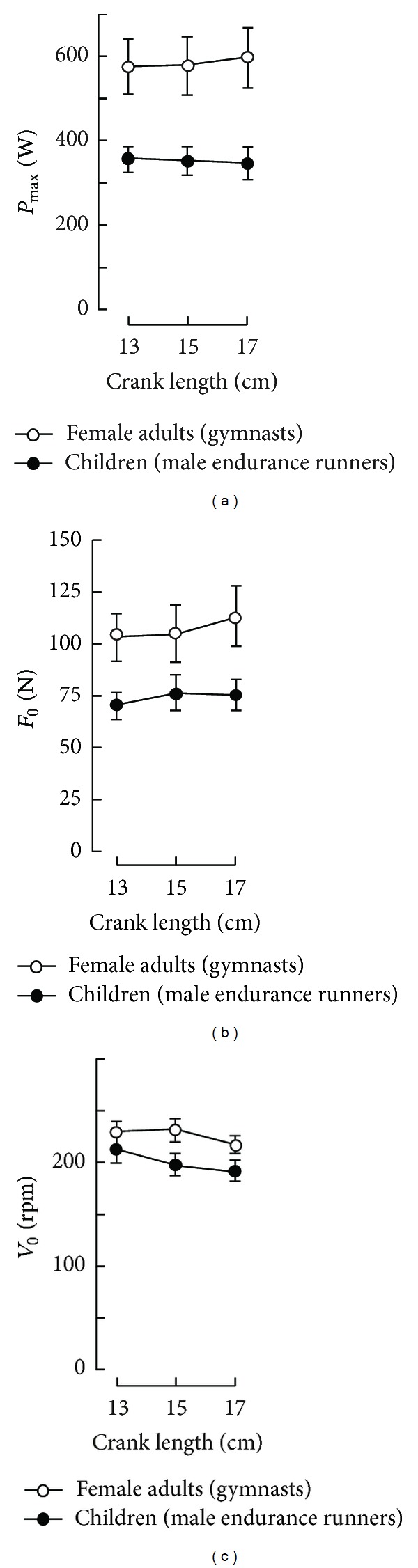
Effects of crank length on parameters *P*_max⁡_, *F*_0_, and *V*_0_ of a force-velocity test on a Monark ergometer in adult female gymnasts and young male endurance runners. Adapted from Vandewalle et al. [81], with permission.

The effects of crank length on performances during all-out cycling exercises were first studied for the Wingate test [148]. Thereafter, the effects of crank length on *V*_0_, *F*_0_, and *P*_max⁡_ were studied in female education students specialised in gymnastic and young male endurance runners [81, 149]. The crank length had opposite effects on *V*_0_ and *F*_0_ (Figure 9), and, consequently, there was no significant effect of crank dimension on *P*_max⁡_. The effects of crank dimension on *P*_max⁡_ and optimal pedalling rate were studied with a larger range of crank lengths (12 to 22 cm) [150]. In this latter study, *P*_max⁡_ was similar with the 145 and 170 mm cranks but was lower with the 120 or 220 mm cranks, in agreement with the results which concerned peak power in the Wingate test [148]. The value of *V*_opt_ decreased significantly with increasing crank length, from 136 rpm (120 mm cranks) to 110 rpm (220 mm cranks) as previously found (Figure 9). In another study by the same research group, the effect of crank length on *P*_max⁡_ was studied with standard 170 mm cranks and a smaller crank length equal to 20% of estimated leg length (LL20) in young boys aged 8–11 years [151]. The value of *P*_max⁡_ with the 170 mm cranks was not significantly different from that produced with the LL20 cranks, but *V*_opt_ was significantly greater with the LL20 cranks.

### 12.3. Cycle Ergometer Design

Simulations using forward dynamics studied the values of crank length, pelvic inclination, seat height, and pedal rate which maximize power output in cycling. In a first study, the value of *P*_max⁡_ given by the simulation was found to be around 1000 W at an optimal pedal rate equal to 135 rpm for a 170 mm crank length and an optimal seat height ranging between 83 and 95% greater trochanter height [152]. In a second simulation, the optimal seat configuration that produced maximum crank power (981 W) corresponded to a higher seat height (102% greater trochanter height) and a seat tube angle of 85° (angle with the horizontal plane) [153]. The influence of seat height was much more important than the influence of seat tube angle (1%) for a wide range of seat tube angles (65 to 110°). However, the influence of seat tube angle on maximal power output was more significant in experimental studies. Peak Power was measured during a 15-second all-out test with seat angles at 69, 76, 83, 90° [154]. Peak power (W) was significantly higher (7.9%) at 69° than at 90°, but the other differences were not significant. In another study, peak power and mean power during a Wingate test were measured during a Wingate test on a Monark ergometer with a backrest, against a braking force equal to 8.5% BW with seat tube angle at −10, 15, 40, and 65° [155]. Peak power (W·kg^−1^BM) at 15° was 7.5 and 11% higher when compared with seat tube at −10 and 65°, respectively. Therefore, the results of this study are in favour of a body position close to recumbent cycling with a backrest. 

### 12.4. Inertial Load

It is possible to increase the resistance to acceleration due to flywheel inertia either by increasing the dimensions of the flywheel or by increasing the ratio between crank angular velocity and flywheel angular velocity (gear ratio). In these cases, resistance to acceleration is high enough without the addition of a frictional resistance, and the torque velocity can be determined for a large range of torques. For example, the resistance was provided solely by the moment of inertia of the flywheel in a study measuring the torque-velocity relationship during a single all-out sprint [125]. The use of the same ergometer in young children as in adults results in an increase of the time necessary to reach *V*_peak_ because of the heavy flywheel inertia. However, this delayed peak corresponds to a small decrease (about 3%) of *P*_max⁡_ in children [156] and cannot explain the large difference in *P*_max⁡_ (W·kg BM^−1^) between children and adults [156, 157].

### 12.5. Eccentric versus Circular Chainring

A circular chainring provides a constant radius from the crank center to the chain driving the wheel. In contrast, the radius of a noncircular chainring varies with crank angle and modifies the crank angular velocity profile over a pedal revolution. A theoretical study focused on the design of noncircular chainrings that maximized crank power suggests that average crank power output can be increased by utilizing a noncircular chainring that allows muscles to generate power for a longer duration during the powerstroke [158]. The corollary of a longer powerstroke is a shorter time at the bottom dead center, that is, the sector corresponding to the relaxation of the muscles active during downstroke. The rates of force development and relaxation can limit the production of torque and power during fast cyclical movements [41–44]. An incomplete relaxation at the beginning of upstroke because of a shortening of the time at the bottom dead center would result in negative work and decrease in cycling mechanical efficiency. Several studies have compared the cycling performances with conventional chainrings and noncircular chainrings. Hue et al. have found better performances in a force-velocity test on cycle ergometer [159]. On the other hand, the interest of noncircular chainrings is not obvious for longer exercises. Significantly higher performances have been observed from the beginning to the 25th second of a Wingate test but not at the end of the test (30th second) [160]. The performance in a simulation of an all-out 1 km event was improved with noncircular chainring [161] on a cycle ergometer in the laboratory, but this result was not confirmed in another study on a 1 km exercise performed on the track [162]. Better mechanical efficiency [163] and delta efficiency [164] with a noncircular chainring have been reported. However, other studies reported no differences between noncircular and circular chainrings for aerobic performance indices [161, 165–167] or even lower performances [162] with the noncircular chainrings. Therefore, it is likely that the use of noncircular chainrings improved performance in all-out sprint by increasing duty cycle but not mechanical efficiency as suggested by the results of studies on long-lasting exercise at lower pedal rates and power outputs. 

In summary, the values of *P*_max⁡_, *V*_0_, *V*_opt_, *F*_0_, and *F*_opt_ depend on the material: (1)  *P*_max⁡_ measured with strain gauge bonded to the cranks (or force transducer in the pedals) is approximately 10% higher than *P*_max⁡_ measured at the flywheel level because of energy losses due to friction in the chain and sprockets; (2) small variations in crank length (±10% around the optimal length) have no significant effect on *P*_max⁡_ because they have opposite effects on *V*_0_ and *F*_0_; (3) the relative importance of seat height and seat tube angle is debatable; however, it is possible that *P*_max⁡_ is maximal with body position close to recumbent cycling with a backrest; (4) the use of a heavy flywheel inertia results in an increase of the time necessary to reach *V*_peak_, which could partly explain the low value of *P*_max⁡_ in children; (5) the use of noncircular chainrings could improve performance in short all-out sprint by increasing duty cycle but not mechanical efficiency.

## 13. Effect of Protocol

In the usual protocol, the Wingate test begins from a rolling start, around 60 rpm, against a low resistance, and then the load is rapidly set. The proponents of a rolling start (between 60 and 100 rpm) assume that this start gives a faster rise to peak power. On the other hand, the standardization of the test is easier with a stationary start, and its reliability should be improved. In spite of a longer time to peak with a stationary start, Peak Power was significantly 11% [168] and 17% higher [169] when compared with a rolling start.

**Figure 10:**
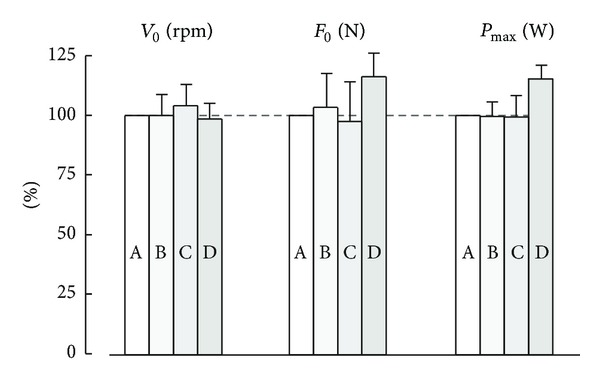
Effects of the protocol on parameters *P*_max⁡_, *F*_0_, and *V*_0_ of a force velocity test on a Monark ergometer. In reference protocol (A), the subjects were seated without restraining belt and the test began with the lowest load. In B, the test began with the highest load. In C, the test was performed with a restraining belt. In D, the subjects were standing up on the pedal. Adapted from Vandewalle et al. [81], with permission.

The effects of the protocol on the force-velocity test (Figure 10) have been studied by comparing a protocol with increasing loads in a seated position without a belt (protocol A) and three other protocols: decreasing load in seated position without a belt (protocol B), increasing load with restraining belt (protocol C), and standing-up (protocol D) [81]. There was no difference between the protocols with increasing (A) and decreasing loads (B) for *P*_max⁡_ as well as *V*_0_ and *F*_0_. In another study *P*_max⁡_ was 9.8% higher in a second session which began with a load equal to *F*_opt_ determined during the first session [170]. However, a training effect between the first and second sessions could not be excluded in this latter study. In the protocol C, a restraining belt was placed around the waist and anchored to the saddle to maintain the seated position, as in the first studies on the isokinetic torque-velocity relationship [24, 110]. Indeed, it was assumed that the body weight might be insufficient to counteract the force exerted on the pedal at high loads and that the subjects could exert their maximal force by pulling against the belt. Unexpectedly, *F*_0_ was slightly lower with a restraining belt, and the only significant difference concerned *V*_0_ which was improved, whereas *P*_max⁡_ was unchanged.

The performances in a 30-second Wingate test were improved by 8% when the subjects stood up on the pedals [171]. It is likely that additional power from the upper body can be transferred across the hip, which could explain the 13 and 15% increases in *P*_max⁡_ and *F*_0_ with the standing-up protocol (D, Figure 10). When compared with similar athletic groups, maximal power was approximately 15% higher in a force-velocity test with a standing-up protocol [172, 173]. For a 3-second inertial load test, the performances were improved by 12% when the subjects stood up on the pedals [174, 175].

Warm-up procedures and previous exercises influence the values of *P*_max⁡_ [176, 177]. In the force-velocity test with a friction-braked ergometer, the sprints with the first and second loads (protocol with increasing loads) must be considered as learning and warm-up exercises and performed again at the end of the test [104, 105]. Indeed, the peak velocity corresponding to the first trial with the first load (1 kg for the arms, 2 kg for the legs) is often 10 rpm higher (unpublished personal data) when compared with the second trial with the same load at the end of the test. The total duration of a force velocity test on a cycle ergometer is approximately 30–40 minutes because of the five-minute recovery intervals between the all-out sprints and the repetitions of the two first loads [104, 105]. The effect of recovery duration between the all-out repetitions (30 s, 1 min, 3 min, 5 min, 10 min, and 24 h) upon *P*_max⁡_ was not significant in an experimental study on physical education students [178]. When compared with the 30-second recovery intervals, the 6% higher value of *P*_max⁡_ with 24 h recovery was not significant. Similarly, there was no significant difference between the other recovery protocols. Another study compared 15, 30, 60, and 120 s recovery intervals between two all-out cycling sprints performed against *F*_opt_ [179]. The values of PP_corr_ and *P*_max⁡_ of the second sprint were significantly lower for the 15 and 30 s recovery but not for the 60 and 120 s intervals. However, the effect of recovery intervals was not studied for more than two repetitions. Moreover, the recovery intervals between all-out sprints should be longer in power athletes who possessed higher percentages of fast muscle fibers, lower aerobic potential, and less developed capillary network (see the chapter on the bioenergetics of all-out prints).

The effects of active recovery (32% of maximal aerobic power) between short all-out sprints have been studied for a force-velocity test with 5-min recovery intervals [180]. Blood lactate at *F*_opt_ (6.73 versus 8.54 mmol·L^−1^) was significantly lower with active recovery which was associated with a significant 7% increase in *P*_max⁡_ (945 versus 883 W). The effect of activity during very short recovery intervals (30 s) was studied in subjects performing eight 6-second all-out tests separated by 30-second recovery either sitting passively on the bike or pedaling at 60 W [181]. The values of peak power were significantly improved by active recovery. This better recovery is attributed to a lowering of muscle lactate. The influence of blood lactate (instead of muscle lactate) on *P*_max⁡_ is probably minor. For example, there was no difference in *P*_max⁡_ in the study on the effects of recovery duration in spite of difference in blood lactate [178]. Similarly, an increase of blood lactate concentration (11.0 mM) induced by a previous arm exercise (5 min of heavy arm cranking) had no significant effect on peak power of a Wingate test performed with the legs (4% drop, *P* > 0.05) [182].

In summary, maximal power depends on the protocol: (1) warm-up procedures improve maximal power (PP or *P*_max⁡_); (2) power indices are improved by 8 to 15% when the subjects stand up on the pedals whatever the test (Wingate test, force-velocity test, pure inertial all-out sprint) probably because additional power from the upper body can be transferred across the hip; (3) active recovery at low intensity improves performance when sprints are repeated; (4) a stationary start should be preferred to a rolling start because performance is not significantly lower, but the standardization of the test is easier and its reliability should be improved.

## 14. Reliability

The reliability of a test is defined as the consistency or reproducibility of performance when someone performs the test repeatedly [183]. The reliability of the results of the Wingate test measured by the test-retest coefficient of correlation is good for the peak power (*r* > 90) [7, 143] and the mean power (between 0.91 and 0.93) [93, 143]. On the other hand, the reliability of the fatigue index is low (*r* = 0.43).

**Figure 11:**
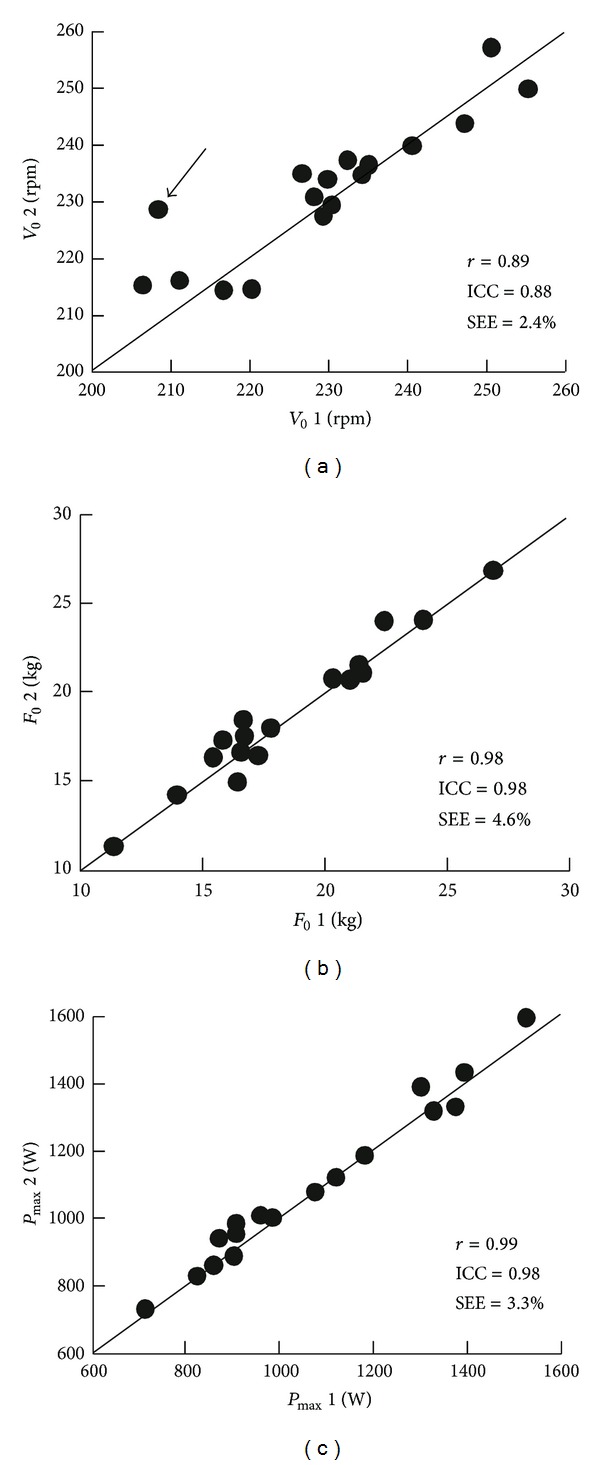
Reliability of the parameters *V*_0_, *F*_0_, and *P*_max⁡_ of a force-velocity test on a Monark cycle ergometer; abscissa and ordinates, results of the first and second sessions, respectively. Lines of identity. ICC, intraclass correlation; SEE, standard error of estimation. Adapted from Attiogbé et al. [184].

The reliability of the force-velocity parameters (*V*_0_, *F*_0_, and *P*_max⁡_) was tested in physical education students (Figure 11) [184]. The values of *r* (test-retest) and ICC were higher than 0.9 and SEE lower than 5% for *F*_0_ and *P*_max⁡_. The correlation coefficients (*r* and ICC) were lower for *V*_0_ because of the smaller variance of this parameter. However, as indicated by the value of SEE (2.4%), the reliability of *V*_0_ was high in all the subjects but one (arrow in Figure 11). This test-retest study was performed after one habituation session and at the same hour for both sessions to limit the time-of-day effect.

The coefficient of variation (test-retest) of the maximal peak torque was lower than 6% in the study by Sargeant et al. on isokinetic cycling [24]. The coefficients of variation of the slope and intercept of the regression between torque and pedal rate on isokinetic ergometer were 13.7 and 10.5%, respectively [110]. In the same study, the coefficient of variation was 8.6% for the peak power of a 30-second all-out isokinetic cycling exercise [110]. In another study on isokinetic torque-velocity relationship, the between-days test-retest correlation coefficient was equal to 0.984 for *P*_max⁡_, and the limit of agreement (95% random error) was 0.0498 ± 0.397 W·kg^−1^ [112].

In physical education students tested five times within 15 days, PP_corr_ measured during session 2 was 4.3% higher than during session 1 (*P* < 0.001) [185]. When the protocol included at least two sprints in adults, the measurement of cycling peak power was found to be highly reliable (test-retest coefficient of variation approximately 3%).

The reliability of the results of the inertial load test has been investigated in two studies [125, 186]. The mean coefficients of variation of the different parameters measured with the inertial method (4 trials on the same day) were 3.3% for PP_corr_, 2.7% for *V*_0_, and 4.4% for *T*_0_ [125]. The intraclass correlation coefficient was 0.99 for the subject's PP_corr_ over the repeated bouts. These results were confirmed in the other study on interday (3-day intervals) and intraday (4 trials with 180-second recovery intervals) reliability of PP_corr_ (*r* = 0.99 for interday and *r* = 0.94 for intraday) [186].

The between-days test-retest correlation coefficient was equal to 0.975 for *P*_max⁡_ measured during a single-bout force-velocity test against a 20 N braking force, and the limit of agreement (95% random error) was 0.0153 ± 0.706 W·kg^−1^ [112]. 

The reliability of power and work indices has also been studied for the repeated-sprint cycling tests (5 × 6 seconds and 4 × 5 seconds). The reliability of the 5 × 6-second cycling test was tested in five sessions [127]. Significant improvements in all the work and power indices were observed between session 1 and subsequent sessions (*P* < 0.05), but no significant differences were identified between sessions 2, 3, 4, and 5. However, there were large variations in decrement indices between sessions, which probably limits the interest of this repeated-sprint cycling test. The reliability of the 4 × 5 second repeated-sprint cycling test was tested for interday variability between 3 sessions [128]. There was no significant difference between the peak 5-second power output, mean power output, and the fatigue index (%) among the 3 different sessions. The intraclass correlation coefficient for peak 5-second power output and mean power output was 0.82 and 0.86, respectively. In contrast with the Wingate test and the other repeated-sprint test (5 × 6 seconds), the reliability of the fatigue index was also high (ICC = 0.82). 

The conclusion of the previous study on 5 × 6 s cycling test reliability was that two familiarisation sessions are optimal for the collection of reliable data. Similarly in a study comparing 6 s sprints on a cycle ergometer on four separate occasions, peak power was significantly higher (4.9%; *P* < 0.05) in trial 2 compared with trial 1, whereas there were no significant differences between trials 2, 3, and 4 [187]. Therefore, it is likely that one familiarisation session is useful when accurate assessments of *P*_max⁡_ or PP_corr_ are needed whatever the method and the test. In young children, the practice of all-out cycling exercises the days before testing is probably necessary [82, 188, 189].

In summary, the reliability of maximal power indices is high, whatever the protocol (Wingate test, force-velocity test, inertial load test, repeated-sprint test) and the ergometer (friction-braked or isokinetic). However, it is likely that one familiarisation session is useful or even necessary in young children. In contrast, the reliability of the fatigue indices (fatigue index of the Wingate test, decrement indices of the repeated-sprint tests) is low even after familiarisation sessions.

## 15. Correlation with Other Laboratory Tests

Significant correlations have been found between maximal power on a cycle ergometre and vertical jump performances [105, 190–194] and the stair case test of Margaria [191]. *P*_max⁡_ expressed per kilogram of body mass is significantly correlated with a squat jump (SJ) [192, 194] and a countermovement jump (CMJ) [105, 193]. However, the prediction of CMJ [105] or SJ [194] from *P*_max⁡_ is not accurate in spite of high correlation coefficients (*r* = 0.87) [105, 192]. For example, individual errors were close to 40%, and the authors concluded that squat jump is recommended in large-scale developmental prospective studies but that cycling and jumping protocols are not interchangeable when measuring peak power [194]. In karate competitors, *P*_max⁡_ in cycling was not significantly correlated (*r* < 0.42) with performances in squat jump and countermovement jump [195].

In volleyball players, CMJ was also significantly correlated with *F*_0_ in cycling [193]. In addition, *V*_0_, *F*_0_, *P*_max⁡_ in cycling were significantly correlated with the same parameters in cranking [193]. In another study, squat jump was also significantly correlated to *V*_opt_ in cycling (*r* = 0.86) [192].

Peak power during an inertial load test is highly correlated (*r* = 0.82) with the peak power of a Wingate test) [186]. However, the peak power during the inertial load test (1268 ± 41 W) was significantly higher (*P* < 0.001) when compared with the peak power of the Wingate test (786 ± 27 W). The mean power during a Wingate test was significantly correlated with the result of the Bosco anaerobic test which consists in the repetition of maximal vertical jumps during 30 seconds [196].

## 16. Correlation with Field Performances in Cycling

In the following lines, it will be assumed (1) that there is no slippage of the wheel on the road; (2) that the rotational kinetic energy of the wheels and the energy loss in the tyres are negligible. The cycling speed (*S* in m·s^−1^) is equal to *VD* the product of pedal frequency (*V* in revolution·s^−1^) and development (*D* in m·rev^−1^). According to the principle of energy conservation, the relationship between *V*, *F*, *S* and the force *F*_Road_ exerted on the road is [197]

(20) $$\begin{array}{c} P=\omega T=S\cdot F_{\text{Road}}=\;V\cdot D\cdot F_{\text{Road}},\\ F_{\text{Road}}=\frac{\omega T}{VD}=\frac{2\pi T}{D},\\ F_{\text{Road}}=\frac{2\pi T_{0}\left(1-\omega /\omega_{0}\right)}{D}\;=\;F_{0\;\text{Road}}\left(1-\frac{S}{S_{0}}\right), \end{array}$$

where *S*_0_ is equal to product *V*_0_ *D* and *F*_0  Road_ is equal to 2*πT*_0_/*D*. As a consequence, the relationship between power *P* and *S* is equivalent to the relationship between power and pedal frequency in laboratory testing:

(21) $$\begin{array}{c} P_{\max}=0.25\omega_{0}\cdot T_{0}=0.25S_{0}\cdot F_{0\;\text{Road}},\\ P={SF}_{\text{Road}}=4P_{\max}\left(\frac{S}{S_{0}}-\frac{S^{2}}{{S_{0}}^{2}}\right). \end{array}$$

**Figure 12:**
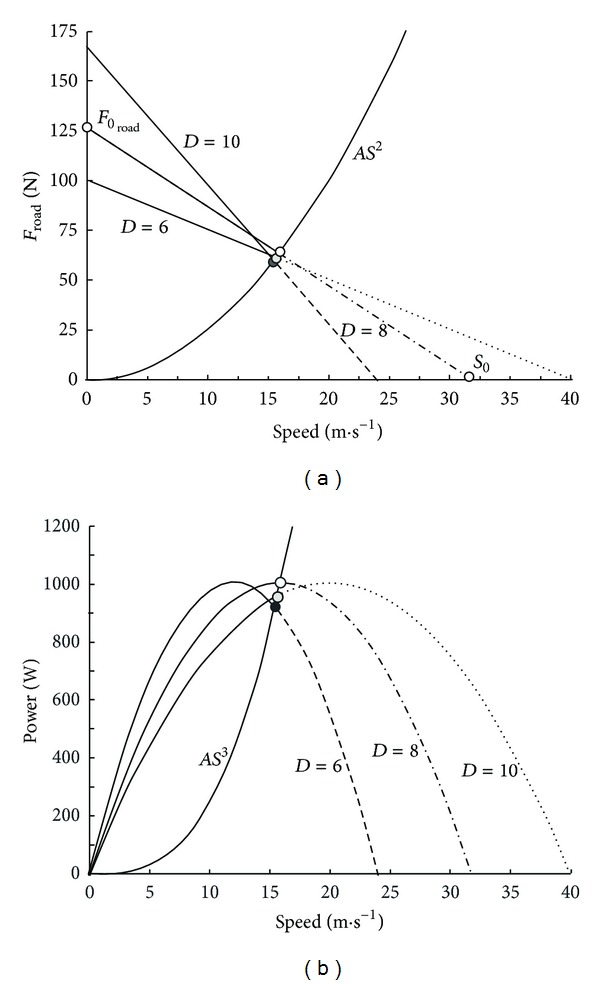
Prediction of the maximal cycling speed from the torque-crank velocity relationship (*ω*_0_ = 25 rad·s^−1^; *T*_0_ = 160 N·m; *P*_max⁡_ = 1000 W) in function of different meters of development *D* (6, 8, and 10 m) for *A* = 0.25.

This relationship is presented on Figure 12 for 3 different values of meters of development (6, 8, and 10) in a subject whose values of *P*_max⁡_ and *ω*_0_ are equal to 1000 W and 25 rad·s^−1^ (4 rev·s^−1^ or 240 rev·min^−1^), respectively. When speed *S* reaches its peak value (*S*_Peak_) during an all-out cycling exercise, acceleration is equal to zero and *F*_Road_ is equal to *R*_Air_. In a first approximation, *R*_Air_ is proportional to the square of speed *S*:

(22) $$\begin{array}{c} F_{\text{Road}}=R_{\text{Air}}=A\cdot S^{2}=F_{0\;\text{Road}}\left(1-\frac{S}{S_{0}}\right),\\ A\cdot {S_{\text{Peak}}}^{2}\;+\frac{F_{0\;\text{Road}}S_{\text{Peak}}}{S_{0}}-F_{0\;\text{Road}}=0, \end{array}$$

where *A*  is a parameter which depends on the frontal area, shape, and air density. Therefore, the value of peak speed corresponds to the positive root *R*_2_ of the following second order equation:

(23) $$\begin{array}{c} A\left[{S_{\text{Peak}}}^{2}+\frac{F_{0\;\text{Road}}S_{\text{Peak}}}{AS_{0}}-F_{0\;\text{Road}}\right]=0,\\ A\left(S_{\text{Peak}}-R_{1}\right)\left(S_{\text{Peak}}-R_{2}\right). \end{array}$$

The maximal speed (*S*_max⁡_) that a cyclist is able to reach is obtained when power output at peak speed is equal to *P*_max⁡_, that is, the velocity corresponding 0.5*V*_0_ *D*. Therefore, *S*_max⁡_ and the optimal value of *D* (*D*_opt_) are given by the following equations:

(24) $$\begin{array}{c} P_{\max}=A{S_{\max}}^{3},\\ S_{\max}={\left(\frac{P_{\max}}{A}\right)}^{1/3}=0.5V_{0}D,\\ D_{\text{opt}}=\frac{2{\left(P_{\max}/A\right)}^{1/3}}{V_{0}}. \end{array}$$

In theory, the relationship between torque and velocity can also be used to predict the cycling speed curve during an all-out sprint on track [197]. The analytic solution of the relation between speed *S* and time *t* corresponds to the following equation:

(25) $$\begin{array}{c} S=R_{1}+\frac{R_{2}-R_{1}}{\left[1-\left(R_{2}/R_{1}\right)e^{-At/Zm}\right]}, \end{array}$$

where *Z* = 1/(*R*_2_ − *R*_1_) and *m* is the mass of the cyclist plus the mass of the cycle.

However, the interest of this equation is limited because it does not take into account the effect of fatigue upon *V*_0_, *F*_0_, and *P*_max⁡_. The validity of the use of the force-velocity relationship for the prediction of field performances in sprint cycling has been verified in a study which compared maximal torque- and power-pedalling rate relationships estimated from the data of an inertial load test and power measured on the field [198]. Torque was measured on the field with an SRM power transducer during 65 m all-out sprints in elite cyclists. There were no statistically significant differences between laboratory and field for *P*_max⁡_ (1791 versus 1792 W), *V*_opt_ (128 versus 129 rpm), and maximum torque (266 versus 266 Nm). As a consequence, the linear regression slope of the torque-pedalling rate was similar (−1.040 versus −1.035) in the field and laboratory tests.

## 17. Biological Factors Determining *P*_max⁡_

The values of *P*_max⁡_ and peak power depend on quantitative and qualitative factor. The muscle mass active during all-out cycling is the main quantitative factors limiting maximal power output. The main qualitative factors are probably fast fiber percentage, mechanical efficiency, and motor control. Moreover, some experimental data indicate that maximal power output depends on fatigue even during the completion of very short all-out exercise. The influences of cycling efficiency, fatigue, muscle mass, percentage of fast muscle fibers, age, and gender as factors limiting maximal power output are discussed in the following paragraphs.

### 17.1. Efficiency

The first assumption underlying the use of power output on a cycle ergometer as an index of aerobic or anaerobic performance is that there is no large difference in efficiency between subjects. The aerobic metabolism provides the energy supply of cycling at low intensity. Therefore, it is possible to compute the mechanical efficiency (work divided by energy consumption) from the measurements of mechanical work and oxygen uptake during these exercises. For example, it was found that the better efficiency in elite cyclists was related to the percentage of type I muscle fibers [199], whereas another study found that there was no significant difference between elite and recreational cyclists [200]. The index of mechanical effectiveness is another approach of the study of efficiency in cycling [201, 202]. The force *F*_*P*_ exerted on the pedal is the sum of a normal component *F*_*N*_ (tangential to the trajectory of the pedal) and a radial component (*F*_*R*_). The index of mechanical effectiveness IE is defined as the ratio of the effective force (*F*_*N*_) to the force *F*_*P*_ exerted at the shoe-pedal interface, that is, ratio *F*_*N*_/*F*_*P*_. It is assumed that a higher value of IE corresponds to a better efficiency.

Differences in cycling efficiency should contribute to the between-subjects variance in *P*_max⁡_·BM^−1^. Unfortunately, the anaerobic metabolism provides the energy supply, and there is no steady state during maximal power output, which makes difficult the measurement of energy consumption and the computation of mechanical efficiency. During an all-out exercise around 120 rpm, the force exerted on the pedal at 90° was almost perpendicular to the crank (IE close to 1), but the index of mechanical effectiveness averaged over a complete cycle was not significantly related to power output [34]. Indeed, in this study, power output at 120 rpm was significantly related to IE during upstroke and top dead sector but not with IE during the total revolution or the downstroke. Therefore, IE was significantly correlated to power output for sectors whose contributions to *P*_max⁡_ were not important, and it is likely that the index of mechanical effectiveness only explains a small fraction of the variance in *P*_max⁡_ [34].

Moreover, the validity of the ratio *F*_*N*_/*F*_*P*_. as an index of effectiveness is questionable [203]. Indeed, the force exerted on the pedal depends not only on the muscle actions but also on the changes in the mechanical energy of the legs (see Appendix B). The changes in the gravitational force are the main component of the changes in leg mechanical energy (Figure 1), and, therefore, the gravitational force is one of the main forces acting on the pedal, especially at low power output. The cyclist cannot modify the vertical direction of this force: the gravitational force is tangential at crank angle equal to 90 and 270° but radial at 0 and 180°. If the components of the pedal force due to muscle actions were purely tangential during the whole revolution, the effectiveness index would be equal to 1 at pedal angles equal to 90 and 270°, only. At a very low power output, which corresponds to low tangential force, the effectiveness index would be low at 0° and 180 because the main component of the force exerted on the pedal would be the gravitational force acting radially. At high power output, that is, high tangential torque, the effectiveness index would be high even at 0 and 180°. Moreover, these nonmuscular, gravitational forces depend on the anthropometry of the subject [204]: the higher is the leg mass, the lower the effectiveness index should be for a given power output. As a consequence, the effectiveness index should be higher in the most powerful subjects when *P*_max⁡_ is related to body mass.

**Figure 13:**
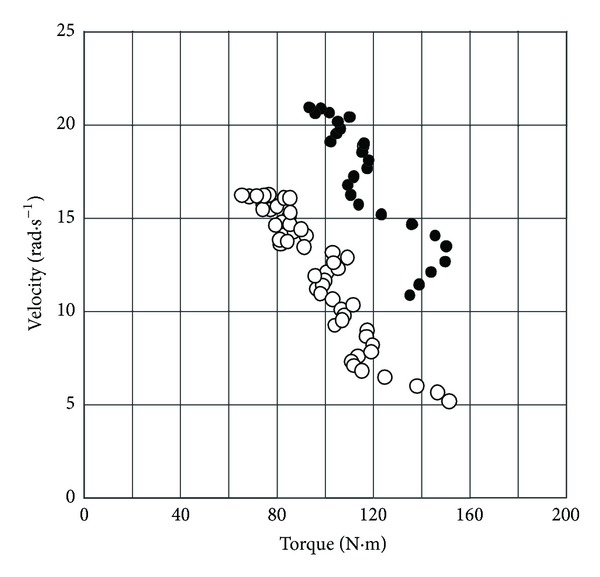
Relationships between crank torque and crank angular velocity during all-out exercises on a Monark cycle ergometer against two braking forces *F*. Empty circles *F* = 8 kg (*T*_*B*_ = 76 N·m); black dots *F* = 2 kg (*T*_*B*_ = 19 N·m) in a subject who had never ridden a bicycle before the test. Adapted from Seck et al. [116], with permission.

It is possible that the control of the cycling movement at high pedal rate by the brain is facilitated by the contribution of spinal Central Pattern Generators [205]. Several studies indicate that shared circuitry could exist in humans and should be seen as a “common core” of CPG elements activated regardless of the specific locomotor task (walking or cycling) [206]. However, it is likely that the practice of all-out cycling exercises is necessary before the assessment of *P*_max⁡_ in young children [82, 188, 189]. For example, maximal power increased 44% from 8.3 to 11.9 W·kg BM^−1^ following 3 days of practice in boys [82, 189]. Similarly, the studies on the reliability of the different indices of maximal power suggest the interest of one or two sessions of familiarization, especially in children. The torque-velocity curves in subjects who never ride a bicycle (Figure 13) indicate that several familiarization sessions are probably needed in these subjects [116]. 

### 17.2. Effects of Fatigue

For cyclic exercise, maximal power output decreases rapidly as the duration of effort increases [207]. The effects of fatigue upon the results of the all-out cycling exercises have mainly been studied for the long-lasting exercises such as the Wingate test. For example, it has been found that the fatigue index equal to the difference between the peak and the lower power outputs during a Wingate test mainly depends on aerobic factors (maximal oxygen uptake, mitochondrial enzymes concentrations, etc.). On the other hand, there are few studies on the importance and origin of fatigue during short (<5 s) all-out cycling exercises.

The lower value of *P*_max⁡_ compared to PP_corr_ in the studies by Seck et al. [116] or Winter et al. [117] was interpreted as a possible effect of early fatigue because the force-velocity test corresponds to peak velocity data instead of data collected during the acceleration phase [116]. Indeed, time to PP_corr_ is approximately equal to 1.5 s [83, 112, 116], whereas time to *V*_peak_ was approximately equal to 3.5 s for all the loads [116]. Time to PP_corr_ increased with the load (0.60, 1.0, 1.5, and 2.0 seconds for the different loads), which could explain why PP_corr_ was lower at high load in the study by Lakomy [113]. Another study carried out on a special cycle ergometer (a 100 kg flywheel ergometer) is probably the only paper which studied the decrease in power output at the very beginning of all-out cycling exercises on a cycle ergometer [147]. The power produced at 0.1 seconds was 19% higher than the power reached at 1.5 second (i.e. time corresponding to PP_corr_) and 35% higher than maximal power at 3.5 s (i.e. time to *V*_peak_). Power output at 1.5 s is 13% higher than at 3.5 s, which is close to the 10–13% difference between PP_corr_ and *P*_max⁡_ determined from a force-velocity test against different braking forces [147]. In theory, maximal power output can be measured during the first revolution of a test performed on an isokinetic ergometer, provided that pedal rate is optimal. Peak power output was reached around 3.3 s at 110 rpm on an isokinetic ergometer [24]. The decreases in torque output during all-out exercises on this ergometer were about 2% per second at 110 rpm [24] and 23% after 10 seconds at 120 rpm [51]. In the curve presented by Kyle and Caiozzo [147], the decrease in power output at 10 s was largely higher (40%) when compared with power output at 0.1 s. However, the decrease in power output at 10 s was similar (about 20%) to Sargeant et al. study when compared with power output at 3.3 s, that is, the time corresponding to peak power in this isokinetic exercise [24]. The fatigue during isokinetic ergometry has been modelled a fourth degree polynomial in one subject [208]. In this study, the maximal slope of power-time curve corresponded to the sixth second of exercise and was equal to −65 W·s^−1^, that is, 4.5% of peak power.

 The torque-velocity relationships corresponding to single all-out sprints against low and high braking forces can be described by the same regression line (black continuous line in Figure 6). However, the regression of the sprint against the heavy resistance (red regression line in Figure 6) was different from the regression corresponding to the sprint against the light resistance (blue regression line). Therefore, the value of *ω*_0_  corresponding to the heavy braking force was lower than the value of *ω*_0_ corresponding to the light force. The torque-velocity data close to *V*_peak_ with the heavy resistance (red empty circle and arrow) in Figure 6 correspond to 4-5% lower values of torque and velocity than the data corresponding to the light resistance (blue empty circle and arrow), that is, 8–10% power decrease.

Isokinetic cycling studies have found that fatigue was greater at high pedal rates (100, 120, 140 rpm) than at a low pedal rate (60 rpm) [74, 209]. Fatigue could be function of the number of cumulated pedal revolutions in addition to the amount of cumulated work and metabolic byproducts [210–212]. Therefore, the effect of fatigue could increase with the duration of the force-velocity tests and the number of revolutions necessary to reach a given pedal rate. The number *N*_*R*_ of revolutions at time *t*  is equal to

(26) $$\begin{array}{c} N_{R}=\int V\;dt=v_{0}\left(1-\frac{F}{F_{0}}\right)\left[t-\varphi \left(1-e^{-t/\varphi }\right)\right],\\ N_{R}=v_{0}\left[t-\varphi \left(1-e^{-t/\varphi }\right)\right]\;\text{for}\;\text{a}\;\text{pure}\;\text{inertial}\;\text{load}. \end{array}$$

Let two loads *F*_1_ and *F*_2_ (*F*_2_ > *F*_1_):

(27) $$\begin{array}{c} N_{R1}=v_{0}\left(1-\frac{F_{1}}{F_{0}}\right)\left[t-\varphi \left(1-e^{-t/\varphi }\right)\right],\\ N_{R2}=v_{0}\left(1-\frac{F_{2}}{F_{0}}\right)\left[t-\varphi \left(1-e^{-t/\varphi }\right)\right],\\ \frac{N_{R1}}{N_{R2}}=\frac{F_{0}-F_{1}}{F_{0}-F_{2}}. \end{array}$$

As the time necessary to reach a given fraction of *V*_peak_ corresponding to *F* is independent of *F* (Figure 5(a)), time *t*_opt_1_ necessary to reach *V*_opt_ with *F*_1_ is shorter than time *t*_opt_2_ necessary to reach *V*_opt_ with *F*_2_ (*V*_peak1_ > *V*_peak2_). At time *t*_opt_1_, *N*_*R*1_ is lower than *N*_*R*2_. Therefore, *N*_*R*_ and *t*_opt_ increase with *F* [116]. This could explain why PP_corr_ decreased as the load increases from 5.5 to 11.5% BW in the study by Lakomy [113]. Interestingly, these effects of fatigue and number of revolutions on the relationship between torque and pedal rate was not observed when torque was measured on the pedal crank with a Lode ergometer in the linear mode [27]. Indeed, the torque corresponding to the peak pedal rate with a high value of *f*_*i*_ (arrows in Figure 8) was not different from the torque corresponding to the same pedal rate at the beginning of a sprint with low value of *f*_*i*_.

Therefore, the magnitude of the fatigue and the importance of the number of revolutions during short all-out cycling exercises are debatable. Moreover, it must be mentioned that some results presented in the study by Kyle and Caiozzo [147] were questionable. The computation of the torque-velocity relationship from the torque-time, velocity-time, and power-time curves presented in this paper gives a hyperbolic torque velocity relationship at velocity lower than 100 rpm and a downward inflection of the torque-velocity curve at velocity higher than 180 rpm because of fatigue. Moreover, the presented data corresponded to one subject, only.

The effect of fatigue could partly explain the lower value of *P*_max⁡_ measured with a friction-braked ergometer in children when the flywheel inertia is not adjusted to body dimensions: time to peak velocity is higher in children with the standard flywheel inertia [156]. However, the fatigue effect due to this delay in peak velocity could only explain a 3% lower value of peak power.

In summary, it is likely that the effects of fatigue upon force, shortening velocity, and power occur at the very beginning of all-out cycling exercises, which could explain the lower value of *P*_max⁡_ compared to PP_corr_. The decrease in power output during all-out exercises at 110–120 rpm is probably about 2% per second. The possibility of a significant fatigue effect at the very beginning (0.1 s) of an all-out exercises suggested in the study by Kyle and Caiozzo is questionable.

### 17.3. *P*_max⁡_ and the Volume of Active Muscles

The determination of the active muscle volume is not only a question of anthropometry but also a question of biomechanics and physiology: are all the muscles producing their maximal power at *P*_max⁡_?; what are the contributions of the different muscle groups in power production during cycling and what are their volumes? The EMG records [32, 33, 35] and functional magnetic resonance studies [213] indicated that most of the leg muscle groups are involved in all-out sprint. Cycling corresponds to a circular movement of the foot, and this movement does not correspond to simultaneous maximal activations of all the leg extensor muscles during downstroke and leg flexor muscles during upstroke. In a same muscle group, the percentages of slow and fast fibers depend on the muscles. For the plantar ankle flexors, slow fibers and fast fibers prevail in the soleus and gastrocnemii, respectively. Therefore, it is likely that pedal rate cannot be simultaneously optimal for power output in all the muscles. Moreover, at high power and/or pedal rate, biarticular muscles can be fully activated without producing power if they had to contract isometrically to be able to produce the force necessary for energy transfer between joints.

The value of *P*_max⁡_ was significantly correlated with the different indirect estimations of the active muscle mass: lean thigh volume (LTV) [82], quadriceps muscle mass [214], thigh tomodensitometric radiograph [83], and lean leg volume [215, 216]. Leg muscle volume should be correlated with lean body mass, which explained that lean body mass was the most important explanatory variable of the variance of *P*_max⁡_ (72%) in obese and nonobese adolescents [80]. The estimated lean thigh volumes of the two legs were 9.8 [24], 10.4 [82], and 12.5 L [214] in young adults, which corresponded to values of *P*_max⁡_ related to thigh volume equal to 85, 133, and 66 W·L^−1^, respectively. As active muscle volume also includes lower leg muscles and monoarticular hip flexors and extensors, *P*_max⁡_ related to active muscle volume should be lower. In a large scale MRI study, the thigh muscle mass (9.38 kg) in young male adults represented approximately 50% of the lower body muscle mass (18.5 kg) measured from one image below L_4_-L_5_ to the foot [217]. Therefore, *P*_max⁡_ related to active muscle volume should be between 33 and 66 W·L^−1^ in a general male adult population.

It is interesting to compare *P*_max⁡_ with recent data on *P*_max⁡_muscle_ measured in single muscle fibers (*P*_max⁡_fiber_). As velocity is highly sensitive to temperature, the differences in *P*_max⁡_muscle_ between the studies on single muscle fibers are very large because of differences in the temperature of the bath and, probably, difference in the accuracy of the determination of the muscle fiber dimensions. *P*_max⁡_fiber_ ranges between 0.3 (human, type I, 12.5°C) [18] and 230 W·L^−1^ (rat flexor hallucis brevis, type II, 35°C) [218]. However, *P*_max⁡_muscle_ corresponds to the product of the instantaneous values of force and velocity during shortening, whereas *P*_max⁡_, peak power, and PP_corr_ in cycling correspond to power output averaged over one revolution, that is, during active shortening and passive lengthening. On an isokinetic cycle ergometer [24], the maximal value of *P*_peak_ (power produced by one leg at the peak of a revolution) was equal to 1387 W for one leg (i.e. 2774 W for two legs), whereas *P*_max⁡_ averaged over one revolution was equal to 840 W for two legs (i.e. 0.3*P*_peak_max⁡_). Similarly, at medium velocity (around 100 rpm), the power output averaged over a revolution and measured at medium velocity (around 100 rpm) during an all-out sprint on a Lode ergometer corresponded to 35–37% *P*_peak_ during the same revolution (Figure 2). Therefore, the ratio *P*_peak_max⁡_/*P*_max⁡_ should be approximately equal to 2.8. A value of *P*_max⁡_ equal to 1000 W at the flywheel level should correspond to a value of *P*_peak_max⁡_ about 2800 W at the flywheel and about 3000 W at the crank level because of frictional loss between the crank and the wheel. Therefore, the value of *P*_peak_max⁡_ related to thigh volume in the previous studies [24, 82, 214] should be close to 255, 400, and 200 W·L^−1^, respectively. When related to active muscle volume, *P*_peak_max⁡_ should be between 100 and 200 W·L^−1^.

The model proposed by Sargeant is an approach of the relation between *P*_max⁡_ and muscle mass [219]. The shortening velocity cannot simultaneously be optimal for the slow and fast fibers which compose a given muscle. The value of *V*_opt_ of a whole muscle is a compromise between the values of *V*_opt_ of its slow and fast fibers. Therefore, the maximal power output of a mixed muscle is lower than the sum of the maximal powers of its slow and fast fibers. Sargeant's model assumes (1) that the ratio of maximal shortening velocities of normal human type I and II fibers is around 1 : 4 [220, 221]; (2) that the fraction of muscle volume corresponding to fast fibers (*μ*_*f*_) is equal to 0.5; (3) that *V*_opt_ in cycling corresponds to 120 rpm. In this model, at *V*_opt_, fast fibers work at 97% *P*_max⁡_fast_fiber_ and their contribution (*ψ*_*f*_) to *P*_max⁡_muscle_ is about 84%. Therefore, fast muscle fibers working at 97% of their maximal power produce about 2550 W (85% of *P*_peak_max⁡_ equal to 3000 W), which corresponds to 11.4 liters of fast muscle fibers for *P*_max⁡_fast_fiber_ equal to 230 W·L^−1^ [218]. As *μ*_*f*_ is equal to 0.5 in this model, *P*_peak_max⁡_ corresponds to 22.8 liters of active muscles. If the effects of early fatigue on the assessment of *P*_max⁡_ were equal to 19% as suggested by Kyle and Caiozzo [147] and if the activation levels are submaximal (<80%) [40] for some muscles (gluteus maximus, hamstring, tibialis anterior, etc.), the active muscle volume corresponding to *P*_peak_max⁡_ equal to 3000 W and *P*_max⁡_fast_fiber_ equal to 230 W·L^−1^ should be close to 28–30 liters, which is higher than the lower-body muscle volume [217].

It is also interesting to compare these data with those of simulation studies focused on the effect of seat tube angle and seat configuration on maximal power output [152, 222]. Indeed, this simulation gives a value of *P*_max⁡_ (1000 W) close to experimental data in a general population. The effects of muscle volume and muscle power were not studied, but the value of *P*_max⁡_muscle_ related to muscle volume can be computed from the muscle characteristics used in the model. The muscles were assumed to behave according to Hill's equation (see Appendix A) with the following parameters: *F*_0_ = 40.10^4^ N·m^−2^; *V*_0_ = 8 fiber lengths per second and *a*/*F*_0_ = 0.25. Consequently, the value of *P*_max⁡_muscle_ related to muscle volume was (Appendix A):

(28) Pmax⁡_muscle=V0·F0[(k2+k)0.5−k]2=3200  W·L−1[0.309]2,Pmax⁡_muscle=306 W·L−1.

This value of *P*_max⁡_muscle_ was much higher than the values of *P*_max⁡_ related to thigh volume or active muscle volume in the previous studies [24, 82, 214] although maximal power output in the study by Yoshihuku and Herzog [152] (1000 W) was comparable to *P*_max⁡_ in a general male adult population. The value of *P*_max⁡_muscle_ (306 W·L^−1^) in this simulation study was assumed to correspond to mixed muscles. The contribution of fast fibers *P*_fast_ to power output is equal to *ψ*_*f*_ *P*_max⁡_muscle_. *P*_fast_ related to fiber volume is equal to *ψ*_*f*_ *μ*_*f*_^−1^ *P*_max⁡_muscle_. If *ψ*_*f*_ = 0.84 and *μ*_*f*_ = 0.5 as in Sargeant's model [24], and if *P*_max⁡_muscle_ is equal to 306 W·L^−1^, *P*_fast_ would be equal to 514 W·L^−1^ in Yoshihuku and Herzog study [152]. As *P*_fast_ is submaximal in Sargeant's model, *P*_max⁡_fast_ would be even higher and largely superior to *P*_max⁡_fast_ data in the literature.

This discrepancy between data collected in isolated muscles and maximal power output in cycling could be explained by

1. a ratio of maximal shortening velocities of normal human type I and II fibers higher than 1 : 4 (see Appendix A) and/or a lower curvature of the force-velocity relationship in physiological temperature and metabolic conditions;
2. an underestimation of maximal power of the single fibers related to volume (W·L^−1^) because of an overestimation of the volumes of the fibers;
3. a higher contribution of the slow fibers to power output even at high pedal rates, but only at the beginning and the end of the leg extension or flexion, that is, when angular velocity is low;
4. an overestimation of peak instantaneous power output by the muscles due to the nonmuscular contribution to torque (transformation of Δ*E*_Leg_ in *W*_crank_; see Appendix B).

Similarly, the value of *F*_opt_ in cycling was significantly related to thigh muscle area determined from tomodensitometric radiographs of both thighs [83] and different strength indices measured in isometric (maximal voluntary force, maximal rate of force development) or isokinetic conditions [79].

### 17.4. *P*_max⁡_ and Percentage of Fast Muscle Fibers

**Figure 15:**
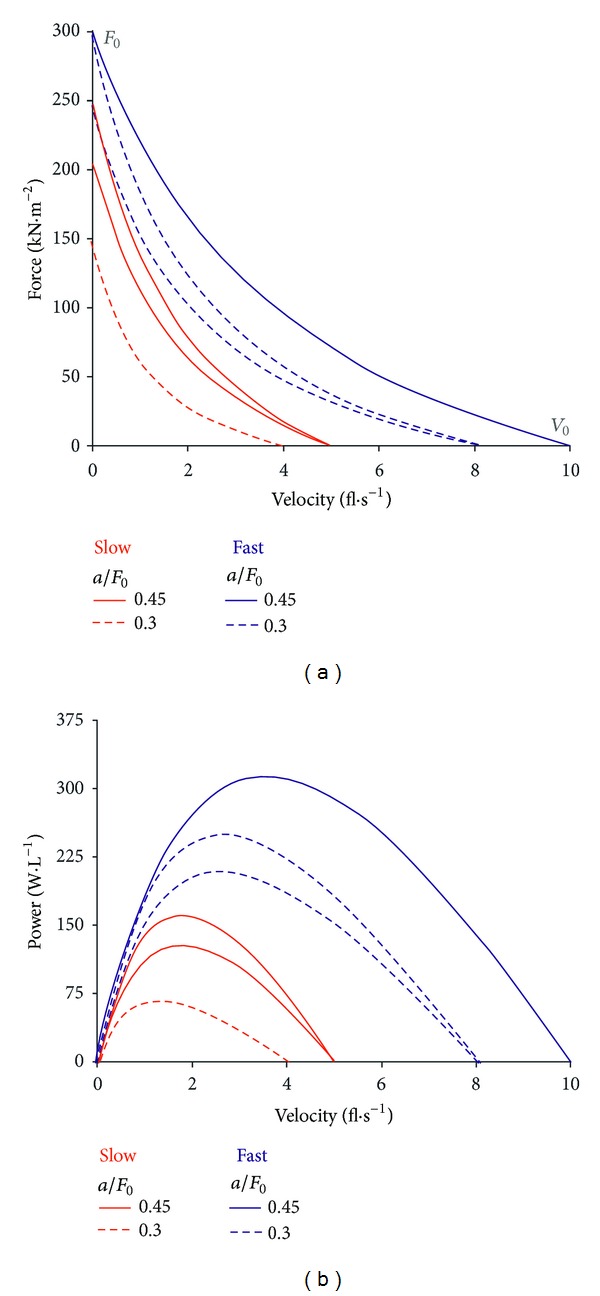
Effects of changes in curvature index *a*/*F*_0_ (0.30 and 0.45), *F*_0_, and *V*_0_ on the force-velocity curves (a) and power-velocity curves (b), according to the hyperbolic model (Hill 1938) in fast (blue curves) and slow (red curves) fibers. Velocity expressed fiber lengths per second (fl·s^−1^).

Power is the product of force and velocity. The maximal power of a muscle fiber mainly depends on its maximal shortening velocity *V*_0_ (see Appendix A). The curvature of the force velocity relationship is the second parameter which determines maximal power: the less curved the relationship is, the higher are the values of *V*_opt_ and *F*_opt_ expressed as fractions of *V*_0_ and *F*_0_. The curvature of the force-velocity relationship is less marked in fast fibers, which partly explain their higher maximal power [15]. Finally, the maximal strains (force/cross-sectional area) of the fast fibers could be slightly higher than those of the slow fibers (Figure 15(a)). The combination of a less curvature and higher values of *V*_0_ and *F*_0_ results in maximal power outputs which are generally considered as much higher in fast fibers (Figure 15(b)). Extreme values of *P*_max⁡_ (from 600 W to 2500 W or from 10 W·kg^−1^ to 25 W·kg^−1^ in male adults) are observed in elite endurance athletes and elite track cyclists [105, 223–225], that is, in the subjects who probably have the lowest and highest proportions of fast fibers, respectively. The relationships between muscle fiber composition and peak power of the Wingate test or *P*_max⁡_, *V*_0_, and *F*_0_ in cycling have been studied from muscle biopsies of the vastus lateralis. The percentage of the fast muscle fibers in the vastus lateralis is significantly correlated with peak power during a Wingate test [226–229] or *P*_max⁡_ [119]. In another study, the correlation between *P*_max⁡_(W·kg BM^−1^) and the proportion of fast twitch fibres expressed in terms of cross-sectional area was close to the significance level (*P* = 0.06) [192].

The value of *P*_max⁡_ is significantly related to optimal velocity [82, 119, 192] or *V*_0_ [193, 195]. *P*_max⁡_ corresponds to optimal pedal rates around 130–135 rpm in best track cyclists [105] and “explosive” athletes [223]. In elite endurance athletes, *P*_max⁡_ corresponds to *V*_opt_ around 100–110 rpm [105]. Consequently, *P*_max⁡_ corresponds to values of *V*_opt_ from 10 to 14 rad·s^−1^. In average, the optimal pedal rate for *P*_max⁡_ is about 120 rpm [105, 223]. *V*_opt_ on an isokinetic ergometer is equal to 110 rpm for an average population [24]. However, subjects with more than 50% of fast twitch fibres reach their maximal power at 119 rev/min and subjects with less than 50% of fast twitch fibres at 104 rpm [230]. The values of *V*_opt_ in subjects with approximately equal proportions of type I and type II fibers in the vastus lateralis were about 120 rpm in another study on isokinetic cycling [111]. The proportion of fast twitch fibres expressed in terms of cross-sectional area was highly correlated to optimal velocity (*r* = 0.88, *P* < 0.001), and the authors of this study suggested that optimal velocity would be the most accurate parameter to explore the fibre composition of the knee extensor muscle from cycling tests [192]. Similarly, *V*_opt_ during sprint cycling was significantly correlated to vastus lateralis MHC-II composition in a study comparing old and young subjects [216].

*P*_max⁡_ was significantly related to *V*_opt_ in a study on maximal power across the lifespan [82]. In a first approximation, lean thigh volume (LTV) is equal to the product of the cross-sectional area and length *λ* of the muscle. *F*_opt_ is significantly correlated to cross-sectional area [83], and, therefore, the product *V*_opt_ LTV should be a function of *λ*, *F*_opt_, *V*_opt_ and, consequently, *λP*_max⁡_. This explained that the product *V*_opt_, LTV was the best predictor of *P*_max⁡_ in the study on maximal power across the lifespan [82].

### 17.5. *P*_max⁡_ and Rate of Force Development Relaxation

*P*_max⁡_ corresponds to values of average crank torque from 65 (endurance athlete) to 150 N·m^−1^ (power athletes). The phase of rising torque exerted on the crank at *P*_max⁡_ (around 110–120 rpm) should last around 0.125–0.150 s as this phase lasts approximately 90–100° during all-out cycling, whatever the pedalling rate [24, 26, 27, 34, 39]. In subjects with high values of *V*_opt_ (125–135 rpm), the phase of rising torque should be shorter. Consequently, in power athletes, high rates of force development are probably necessary to produce high values of torque and power during cycling. The rate of force or torque development and relaxation depends on many factors such as muscle-fibre type, activation-deactivation dynamics, and musculotendinous stiffness. The rate of force development depends on muscle-fibre type: the difference in the rate of force development by single muscle fibres in humans is similar to the difference in their maximal shortening velocities [18], that is, several times higher in fibres IIX than in fibres I. Fast and intense muscle activation is necessary for fast rates of force development [231] and probably not only for high pedal rates [43] but also for maximal power output in cycling as suggested by a simulation of all-out cycling [44]. The rate of force development also depends on musculotendinous stiffness. High musculotendinous stiffness should facilitate not only torque development but also relaxation in the most powerful subjects. Indeed, the musculotendinous stiffness of the ankle plantar flexors measured by quick releases is significantly correlated with *P*_max⁡_ [232]: the higher stiffness was observed in the most powerful subjects.

### 17.6. Effect of Gender, Childhood, and Aging

#### 17.6.1. Effect of Gender

*P*_max⁡_ depends on muscle hypertrophy and muscle fiber types. The cross-sectional areas of all three major fiber types are larger in men [233]. The vastus lateralis muscle contained the same percentage of the different types of muscle fibers [233] in men and women: approximately 41% I, 31% IIA, 20% IIB, and 8% intermediate fibers (1% IC, 1% IIC, 6% IIAB). But there are differences in the cross-sectional areas of the main fiber types: IIA > I > IIB in the men but I > IIA > IIB in the women [233–235]. Consequently, the percentage of the cross-sectional area that corresponds to the slow fibers is significantly higher in women. In another study [236], women have a significantly (*P* < 0.005) higher type I area distribution than men both before (45.0 versus 35.1%) and after (41.9 versus 31.4%) a resistance training program. According to a review study in nontrained young adults, type IIA fibers are generally significantly larger than the other fiber types in men, whereas type I and/or IIA muscle fibers are generally the largest in women [235].

The combined effects of lower fiber size and a higher type I area distribution probably explain the lower values of peak power [237–239] or *P*_max⁡_ in women [80, 240]. When expressed as absolute values (watts), peak power of a Wingate test is significantly lower in women. The difference between men and women was 51, 17, and 5% when peak power was expressed as W, W·kg^−1^ and W·kg^−1^ LBM, respectively [239]. In contrast with power expressed as W or W·kg^−1^, the difference in peak power related to lean body mass (W·kg LBM^−1^) between genders was not significant. In another study, absolute peak power was 35% higher (*P* < 0.001) in men than that in women [238]. This difference was only 10% when peak power was related to kg LBM. Anthropometric variables explained less than 50% of the variation in peak power in men, while in women, thigh volume accounted for 66% of the variation in peak power [238]. When compared with male subjects of the same age, the values of *P*_max⁡_ in female subjects are about 85% at 12 years and 70% at 18 years. The values of *P*_max⁡_ (or peak power) are not significantly different in boys and girls before puberty, but the differences between male and female subjects become significantly different beyond the beginning of puberty whatever the expression of power output (W or W·kg^−1^ BM). After allometric scaling for body mass, men remained more powerful than women for the arm cranking *P*_max⁡_ but not leg cycling *P*_max⁡_ [241].

#### 17.6.2. Maximal Power Output in Childhood

The values of *P*_max⁡_ or peak power in children are significantly lower than in male adolescents and in male young adults whatever the expression of the results (W, W·kg^−1^ BM, W·kg^−1^ LBM). This effect of age upon the maximal mechanical power contrasts with the age effect on maximal oxygen uptake related to body weight, which does not change from childhood to young adulthood in males. The same effect was observed in cross-sectional studies on the effect of age upon the results of other tests of maximal mechanical power such as the Margaria staircase whatever the expression (W, W·kg^−1^ BM, W·kg^−1^ LBM), the gender or the ethnic origins (African, European, or American). Most of the studies on the age effect used 30-second all-out tests derived from the Wingate test [90, 91, 242, 243]. The performances in the Wingate test reach their highest values at the end of the third decade [242].

The same increase from childhood to adulthood has been observed from *P*_max⁡_ measured by means of short all-out cycling exercises [244] or force-velocity tests [82, 215, 245, 246]. The effect of age upon *P*_max⁡_ and peak power was also observed for the exercises performed with the arms. When the Wingate test is performed with the arms (cranking exercise), the highest values are observed at the end of the second decade [242]. In parallel with the improvement in vertical jump (an index of maximal leg power), *P*_max⁡_ (W·kg^−1^ BM) in cranking increases with age in young swimmers who did not practice strength training [247].

The increase in *P*_max⁡_ or peak power of the Wingate test is especially marked during the puberty. The value *P*_max⁡_ of the arms estimated from a force-velocity test in cranking largely increases between 12 and 18 years in parallel with the performance in a countermovement vertical jump [247]. In an allometric transversal study on young male basketball players (13.9–15.9 years) a positive influence of chronological age on the Wingate test was significant even if body size variables were taken into account [91]. In another longitudinal allometric study in children and adolescents (12–17 years), the effect of pubertal maturation on the Wingate performances was not significant in multiple regressions including body mass, fat mass, body height, and gender if chronological age was also included in the multiple regression [90]. However, the braking force (7.5% BW) used in this large scale longitudinal study was probably adjusted to the young children but not to the most powerful adolescents whose performances were possibly underestimated. The increase in *P*_max⁡_ with age and puberty is also observed in female subjects. *P*_max⁡_ increased significantly with body mass, fat-free, mass and lean leg volume in prepubescent girls, adolescent girls, and young women who performed all-out sprints on a friction-loaded cycle ergometer [245, 248]. In growing females, *P*_max⁡_ is primarily dependent upon body dimensions but increases even after correction for lean body mass. This suggests that other undetermined factors, in addition to the amount of lean tissue mass, may explain the increase of peak power and *P*_max⁡_.

The influence of the ergometer (crank length, inertia of the flywheel) is not the main factor explaining the low value of *P*_max⁡_ in young children. The effects of crank length on *V*_0_ and *F*_0_ are opposite (Figure 9), and, therefore, the effects of crank dimension on *P*_max⁡_ are small and not significant [103, 149–151]. The use of the same flywheel in young children results in an increase in time-to-*V*_peak_ when compared with time-to-*V*_peak_ in adults, which should increase the effect of fatigue. This delayed peak could explain a small decrease (about 3%) of *P*_max⁡_ in children [156] but not the large difference in *P*_max⁡_ (W·kg^−1^ BM) between children and adults [156, 157].

There are few studies on muscle fibers in children [234, 249–251]. The percentage of type I fibres in vastus lateralis muscle is about 40% at birth and increases to about 60% within the first two postnatal years [234]. Thereafter, this percentage remains constant, and, for example, there is no significant difference in the fiber type distribution patterns between 6-year-old children and adults [249]. The mean diameter of muscle fibers is about 10–12 micron at birth and increases to 40–60 micron at age 15–20 years [234]. This increase in diameter corresponds to a mean increase in cross-sectional area by a factor of 25. Before the age of 15 years, there is no difference between muscles from males and females, and type I fibres are usually thicker than type II fibres. However, cross-sectional area of type II fibres increases by a factor of 31 in male subjects. Therefore, type II fibers become thicker than type I fibres in male subjects at 20 years. It has been suggested that age-related differences in maximum power production could be also due to differences in intermuscular coordination [246]. For example, the practice of all-out cycling exercises the days before testing is probably necessary in young children [82, 188, 189]. It is also possible that there are differences in the distribution of the individual joint power contributions to total pedal power between adults and children because of their small body size [252]. Indeed, the relative contribution of ankle power to pedal power in children was only half that of adults, and not a significant increase in the contribution of knee joint power was observed in these small subjects.

 According to some data in the literature, age has little or no influence on *V*_opt_ in children [215, 253]. However, *V*_opt_ depends on crank length (Figure 8). Consequently, *V*_opt_ should depend on age in children if crank length is not adjusted to body dimensions. *P*_max⁡_ related to the product *V*_opt_ LTV was stable during the lifespan [82]. Unfortunately, the crank length was not adjusted to the body dimensions of the subjects, and the value of *V*_opt_ was probably underestimated in small and young subjects.

#### 17.6.3. Maximal Power and Ageing

Muscle mass increases with growth up to adulthood and decreases during the last decades (sarcopenia). On average, maximal voluntary strength decreases by 20–40% at 70–80 years, in both men and women, for proximal as well as distal muscles [254]. Loss of muscle mass is the main factor contributing to strength decline in older men and women. The decrease in muscle mass with ageing is the consequence of reductions in fibers size and muscle fiber number. Histological data, from needle biopsy of the vastus lateralis muscle, indicate that the percentage of the different muscle fibers are probably not modified with aging but that average type II fiber size decreases with age, whereas the size of type I fibers is much less affected. Type I fiber area reductions range from 1 to 25%, whereas area reductions range from 20 to 50% in type II. Whole muscle cross-sections from the vastus lateralis muscle obtained on cadaver showed similar reductions in the number of type I and II fibers with aging: 50% fewer type I and type II fibers at the ninth decade when compared with the vastus lateralis muscles from 20-year-old subjects [255]. It is likely that motoneuron losses may be responsible for age-related loss of muscle fibers as suggested by signs of progressive denervation-reinnervation processes secondary to chronic neuropathies (fiber type grouping, fiber atrophy, coexpression of myosin heavy chain isoforms). Moreover, some studies indicate that in aging and disuse, the properties of a muscle fiber type could change with no change in its myosin isoform content [256].

The preferential type II atrophy and the decrease in the total number of muscle fibers with aging result in a decrease in *P*_max⁡_ in older men and women [82, 86, 257, 258]. Maximal power output (*P*_max⁡_, peak power, PP_corr_) significantly decreases after the fourth decade [82, 214, 242]. The decline in *P*_max⁡_ across the adult life span (about 10-11% per decade) is greater than the usually observed decline in maximal leg strength [82, 214]. The declines in *V*_opt_ (3.5–6.6% per decade) and lean thigh volume (LTV) or quadriceps volume (3–5% per decade) confirm that the decline in *P*_max⁡_ is the consequence of a decrease in the fraction of the cross-sectional area corresponding to fast muscle fibers in addition to a decrease in muscle volume. *P*_max⁡_ on a friction loaded cycle ergometer and the corresponding optimal pedal rate (*V*_opt_) were measured in 37 healthy old men (71.1 ± 3.8 years), in 16 young men (22.7 ± 3.4 years) [258], and 29 healthy women (66–82 years) [86]. There were negative relationships between age versus *V*_opt_ or *P*_max⁡_ expressed as W·kg BM^−1^ or *P*_max⁡_ expressed as W · kg_quad_^−1^. From youth to advanced age, *P*_max⁡_W·kg BM^−1^, *P*_max⁡_W · kg_quad_^−1^, *V*_opt_, and quadriceps muscle mass declined in men by 8.3, 5.9, 4.3, and 3.8% per decade, respectively. In women, a multiple stepwise regression analysis showed that mean habitual daily energy expenditure contributed significantly to variance in *P*_max⁡_·kg^−1^, whereas sports activity contributed to variances in *P*_max⁡_W · kg_quad_^−1^ and *V*_opt_. In contrast with women, age was the only variable in men that contributed significantly to variance in *P*_max⁡_.

In summary, the effects of gender, childhood, and aging upon maximal power are mainly explained by the differences in muscle volume and type II fiber size. Maximal power indices are significantly lower in female, children, and aged people when they are compared to male adults even when these indices are related to body mass. These differences are less important when maximal power is related to lean body mass to take into account the difference in fat mass. However, maximal power is higher in male adults even when it is related to active muscle mass. Indeed, muscle power also depends on muscle fiber types. Needle biopsies of the vastus lateralis muscle indicate that the percentages of the different muscle fibers are probably not different but that the average type II fiber sizes are lower in children, female adults, and aged people when compared with male adults.

### 17.7. Effect of Muscle Temperature

The effect of muscle temperature on the indices of maximal or mean power in all-out cycling (PP, PP_corr_, *P*_max⁡_, MP) can be studied after warm-up exercises, changes in environmental conditions, or immersion in water bath. The performances of 30 s all-out sprints performed in a normal environment (18.7 ± 1.5°C, 40% relative humidity) were compared with the same exercises performed in a hot environment (30.1 ± 0.5°C, 55% relative humidity) [259]. When the all-out sprints were performed in the heat, PP_corr_ was about 25% higher (910 versus 656 W; *P* < 0.01) and MP 15% higher (634 versus 510 W; *P* < 0.05). However, PP_corr_ in normal environment was low and probably underestimated as suggested by the faster rate of fatigue (*P* < 0.05) in the hot environment. It is also possible that the “normal” environment was cold in some sessions (18.7 ± 1.5°C) because there was a discrepancy between the temperature in normal environment in the text and figure (18.7 versus 19.7°C). On the other hand, in another study, there was no significant difference in PP when the Wingate test was performed in three different environmental conditions [neutral (22-23°C, 55–60% relative humidity), hot-dry (38-39°C, 25–30% RH), and warm-humid (30°C, 85–90% R.H.)] [260].

Following 45 min of leg immersion in water baths at 44, 18, and 12°C, muscle temperature (*T*_*m*_) measured at 3 cm depth was, respectively, 39.3, 31.9 and 29.0°C, whereas it was 36.6°C without immersion [261]. When compared with pretest rest in the air at ambient temperature, peak power at 95 rpm on isokinetic cycle ergometer increased by approximately 11% after leg immersion at 44°C (i.e., a 2.7°C increase in *T*_*m*_). On the other hand, peak power at the same pedal rate (95 rpm) decreased by approximately 12% and 21% after leg immersion at 18 (4.7°C decrease in *T*_*m*_) and 12°C (7.6°C decrease in *T*_*m*_), respectively. Moreover, the magnitude of the temperature effect was velocity dependent. Peak power increased by approximately 2% per °C *T*_*m*_ when power output was measured at 54 rpm (instead of 95 rpm). When measured at 140 rpm, peak power increased 10% per °C [261]. 

### 17.8. Time of Day and Maximal Power

In a first study, twelve subjects performed the Wingate test on 12 separate occasions (duplicate measurements at 02:00, 06:00, 10:00, 14:00, 18:00, and 22:00 h) [262, 263]. There was no significant effect of time of day upon PP and MP in spite of a temperature peak about 18:00 h (peak to trough equal to 0.76°C). In contrast, the more recent studies on the effects of time of day on short-term exercise indicate that, in neutral environment, the diurnal increase in body temperature (acrophase in the late afternoon) has a passive warm-up effect which improves muscle force and power [264]. Indeed, several studies have observed simultaneous increases in central body temperature and indices of muscular power [264–268]. PP was significantly higher (about 8%) in the afternoon than in morning [265, 269, 270]. Similarly, significant circadian rhythms were found for the results of a force-velocity test on a cycle ergometer [269]. The amplitudes of circadian rhythms were 3.7, 7.0, and 6.9% for *V*_0_, *F*_0_, and *P*_max⁡_ with an acrophase around 18:00 h for *P*_max⁡_. The effect of the interaction of time of day and environment on *P*_max⁡_ was studied in the neutral and moderately warm conditions (20°C and 70% humidity versus 29°C and 57% humidity) [271]. The moderate increase in ambient temperature blunted the diurnal variation in muscular performance, and the improvement in *P*_max⁡_ was significant only in morning. Another study compared the interaction of the time of the day (08:00 versus 18:00 h) and the duration of the warm-up (5 versus 15 min) upon the Wingate test [272]: PP and MP were significantly higher in the afternoon with both warm-up durations. However, the effects of a 15-min warm-up were significantly higher than the effects of a 5-min warm-up in the morning but not in the afternoon. Consequently, longer warm-up protocols are recommended in the morning to minimize the diurnal fluctuations of anaerobic performances.

The effects of time of day were also studied for the ability to repeated sprints [136, 137]. In both studies, subjects performed the same protocol: before starting the RSA test, participants performed a pretest warm-up consisting of 5-min cycling at 84 W and a 10 s maximal sprint test separated by 3-min of rest. Thereafter, the subjects rested for 5 min before performing the RSA cycling test (5 × 6 s maximal sprint every 30 s). In the first study, power output during the first sprint was 5.3% higher in the evening when compared with morning test. But the results of the 2nd to 5th sprints were equal in the morning and evening tests [136]. These results suggested that the increase in muscle temperature following the first sprint “cancelled” out the passive warm-up effect of the diurnal increase in central temperature on subsequent sprints. In the second study [137], power output was significantly higher during the first three sprints in the evening when compared with the morning. In addition to the measurement of power output, surface electromyography (EMG) was collected in four muscles (vastus medialis, rectus femoris, vastus lateralis, and biceps femoris), and neuromuscular efficiency (ratio between work production and muscle activity level) was computed during the five sprints. There was no difference in neuromuscular efficiency between morning and evening tests. Therefore, the diurnal improvement in muscle power and fatigue was interpreted as an improvement of the muscle contractile properties in the evening without a modification in neural drive.

## 18. Conclusions

Power output (peak power) measured at the peak velocity (*V*_peak_) of an all-out test performed on a friction-braked ergometer depends on the braking force *F*. In theory, peak power is maximal for an optimal braking force (*F*_opt_), but given the second order equation between force *F* and peak power, the influence of *F* on peak power is low for *F*_opt_ + 10%. The interest of all-out tests lasting more than 10 seconds is questionable as the mean power and fatigue indices (difference between peak power and the lower power output) largely depend on aerobic metabolism. Therefore, the all-out tests lasting 30 seconds (e.g., the Wingate anaerobic test) should be replaced with short (5 seconds) all-out tests against different braking forces with 5-minute recovery as proposed by Pirnay and Crielaard in 1979 [10]. However, it is likely that this short all-out test cannot be considered as purely alactic. In addition to peak power, the force-velocity relationship in cycling can also be determined by measuring the force exerted on the pedal during all-out exercises on an isokinetic cycle ergometer at different constant pedal rates. The force-velocity relationship in cycling can also be determined indirectly from the acceleration of the ergometer flywheel or directly from the measurement of torque during a single all-out exercise. The force-velocity relationship in cycling is linear between 30 and 200 rpm whatever the type of cycle ergometer (friction-braked or isokinetic) and the protocol (single versus multiple all-out tests). The maximal power output *P*_max⁡_ and the optimal velocity (optimal pedal rate) for power output (*V*_opt_) can be determined from this force-velocity relationship. It is possible that fatigue occurs early at the very beginning of an all-out test, which could explain that the maximal value of power output taking into account the energy necessary to increase the flywheel kinetic energy (PP_corr_) is 10–15% higher than the indices of maximal power output computed from data collected at peak velocity (peak power or *P*_max⁡_). Maximal power depends not only on muscle mass but also on *V*_opt_ which, in turn, depends on the percentage of fast fibers in the leg muscles. The value of *V*_opt_ is about 120 rpm in a general population, whatever the protocol and the ergometer. However, *V*_opt_ varies between 100 and 135 rpm in endurance and power athletes, respectively. *P*_max⁡_ is independent of crank length in contrast with *V*_opt_. The reliability of the different indices of power output (*P*_max⁡_, PP, PP_corr_) is high provided that the all-out exercise measurement is preceded by a habituation session and a minimal warm-up procedure to limit the time-of-day effect. When compared with young male adults, maximal power output related to body mass is lower in prepubertal children, women, and aged people, probably because of a lower muscle volume and a lower relative importance of the cross-sectional area of the fast fibers. The comparison of maximal power output in cycling with data collected on isolated muscle fibers suggests that maximal power (W·L^−1^) is underestimated in single fibers studied at 30°C.

## Figures and Tables

**Figure 14:**
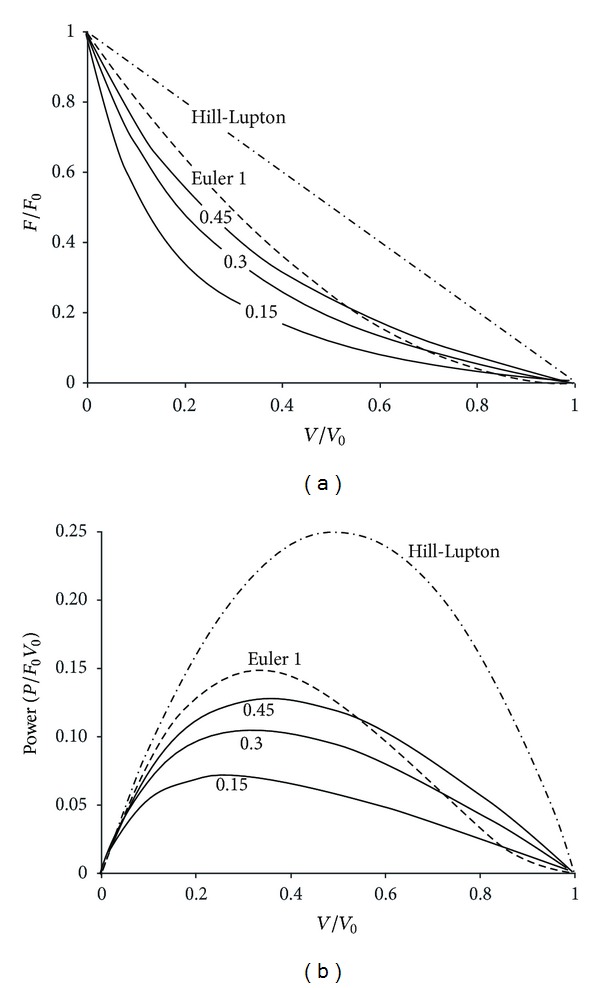
(a) Dimensionless force-velocity relationships of isolated muscle according to the hyperbolic model (Hill 1938) for different values of *a*/*F*_0_ (0.15, 0.30, and 0.45), the linear model of Hill-Lupton (1922) and the second order equation proposed by Euler  ((Euler  1)). (b) Velocity-power curves corresponding to these force-velocity models.

## Appendices

### A.

#### A.1. Relationships between Force and Velocity

The topics of the first studies on muscle properties were not related to mechanics (force, velocity, power) but energetics (maximal work, efficiency of muscular work, metabolism) for the isolated muscle as well as man. According to Amar, the most famous geometers and physicists (Bernoulli, Euler, Coulomb, Coriolis) studied the maximal work in a theoretical way with the method used by the hydraulicians [273]. These scientists imagined that a fluid circulates in the muscle with a velocity *v*, and they assume that the efforts are proportional to the square of *v*. For example, Euler proposed the following formula [273]:

$$\begin{array}{cc} \left(\text{Euler}\;1\right) & F=F^{\prime }{\left(1-\frac{v}{v^{\prime }}\right)}^{2} \end{array}$$

or

$$\begin{array}{cc} \left(\text{Euler}\;2\right) & F=F^{\prime }\left(1-\frac{v^{2}}{{v^{\prime }}^{2}}\right) \end{array}$$

with F′ and *v*′ being the highest effort and the velocity that “make any work impossible.”

In these equations, *F*′ and *v*′ have dimension of a maximal isometric force and maximal shortening velocity in unloading condition, respectively. On Figure 14, the force-velocity relationship corresponding to the first Euler's equation is compared with the force-velocity relationships that have been proposed later.

More than one century later, a force-velocity relationship was deduced from experimental studies on the relationship between maximal work and contraction time in man. For the flexion of the arm in the supinated position, it was found experimentally by Hill [274], and confirmed by Lupton [275], that the work done increases as the speed of movement decreases, according to the formula:

(A.1) $$\begin{array}{c} W=W_{0}\left(1-\frac{\alpha }{t}\right)\;=W_{0}-\frac{W_{0}\alpha }{t}, \end{array}$$

where W_0_ and *α* were constants, and *t* the time occupied in the movement. Hill came to the conclusion that a muscle could be represented mechanically by a spring working in a viscous medium. For Hill, The external work done in a muscular contraction is diminished through viscosity by an amount depending upon the velocity of shortening. *W*_0_ was a constant corresponding to the theoretical maximum work and *α* another constant probably depending on the viscous resistance of the muscle to a change of form. As the amplitude of the elbow flexion (*L*) was the same for all the loads, the work-time relationship corresponded to force-time relationship:

(A.2) $$\begin{array}{c} W=W_{0}\left(1-\frac{\alpha }{t}\right)=W_{0}-\frac{W_{0}\alpha }{t}\;=LF={LF}_{0}\left(1-\frac{\alpha }{t}\right),\\ W={LF}_{0}\left[1-\frac{\left(\alpha /L\right)}{\left(t/L\right)}\right],\\ F=F_{0}\left[1-\frac{\left(\alpha /L\right)}{\left(t/L\right)}\right]=F_{0}\left(1-\frac{V}{V_{0}}\right). \end{array}$$

Ten years later, several force-velocity relationships were proposed from data collected “*in vitro”* on electrically stimulated isolated muscles instead of “*in vivo*” voluntary contractions in man. For Fenn and Marsh [12] or Aubert [276], the force velocity relationship was exponential:

(A.3) $$\begin{array}{c} F=F_{0}e^{\left(-V/B\right)}-KV, \end{array}$$

$$\begin{array}{cc} \left(\text{Aubert}\right) & F=Ae^{\left(-V/B\right)}-K. \end{array}$$

In 1938, Hill [13] proposed a hyperbolic relationship between force and velocity:

(A.4) $$\begin{array}{c} \left(F+a\right)\left(V+b\right)=b\left(F_{0}+a\right)=a\left(V_{0}+b\right)=\text{constant}, \end{array}$$

where *F*_0_ is the maximal isometric force (i.e., the force corresponding to zero velocity), *V*_0_ the maximal velocity (i.e., the velocity corresponding to zero force), *a* and *b* parameters that have the dimensions of force and velocity, respectively. The data of the force-velocity relationships of isolated muscles or skinned fibers in the literature are generally expressed as fiber lengths per second (fl·s^−1^) for *V*_0_ and related to cross-sectional area (kPas or kN·m^2^) for *F*_0_. Hill's force-velocity equation is generally used in the simulation studies on cycling optimisation [152, 222].

#### A.2. Dimensionless Hill's Relationship (Figure 14)

Let *f* = *F*/*F*_0_; *v* = *V*/*V*_0_, and *k* = *a*/*F*_0_ = *b*/*V*_0_. The force velocity can be written with dimensionless variables (*f*,  *v*, and *k*):

(A.5) $$\begin{array}{c} \left(f+k\right)\left(v+k\right)=\left(1+k\right)k,\\ f=\frac{k\left(1-v\right)}{\left(v+k\right)}=\frac{\left(1-v\right)}{\left(1+v/k\right)},\\ v=\frac{k\left(1-f\right)}{\left(f+k\right)}=\frac{\left(1-f\right)}{\left(1+f/k\right)}. \end{array}$$

When 1/*k* = 0, the force-velocity relationship is linear:

(A.6) $$\begin{array}{c} f=\left(1-v\right),\\ F=F_{0}\left(1-\frac{V}{V_{0}}\right),\\ V=V_{0}\left(1-\frac{F}{F_{0}}\right). \end{array}$$

Let *p* = *P*/*V*_0_ *F*_0_

(A.7) $$\begin{array}{c} p=vf=\frac{k\left(v-v^{2}\right)}{\left(v+k\right)}. \end{array}$$

The maximum of *p* corresponds to *dp*/*dv* = 0

(A.8) $$\begin{array}{c} \frac{dp}{dv}=-k\left[v^{2}+2kv-k\right]=0. \end{array}$$

Hence, the velocity and force corresponding to *P*_max⁡  muscle_ (*V*_opt_ and *F*_opt_) correspond to the positive root of the second order equation [*v* ^2^ + 2*kv* − *k*]:

(A.9) $$\begin{array}{c} v_{\text{opt}}=f_{\text{opt}}=\left[{\left(k^{2}+k\right)}^{0.5}-k\right],\\ p_{\max\;\text{muscle}}=v_{\text{opt}}\cdot f_{\text{opt}}={\left[{\left(k^{2}+k\right)}^{0.5}-k\right]}^{2},\\ F_{\text{opt}}=F_{0}\left[{\left(k^{2}+k\right)}^{0.5}-k\right],\\ V_{\text{opt}}=V_{0}\left[{\left(k^{2}+k\right)}^{0.5}-k\right],\\ P_{\max\;\text{muscle}}=p_{\max\;\text{muscle}}V_{0}\cdot F_{0}=V_{0}\cdot F_{0}{\left[{\left(k^{2}+k\right)}^{0.5}-k\right]}^{2}. \end{array}$$

*k*  ranges between 0.25 and 0.30 in fast muscles [15]. If *k* = 0.30,

(A.10) $$\begin{array}{c} v_{\text{opt}}=f_{\text{opt}}=0.3245,\\ p_{\max\;\text{muscle}}=f_{\text{opt}}v_{\text{opt}}=0.105. \end{array}$$

In slow muscles [15], *k* ranges between 0.15 and 0.17. If *k* = 0.15,

(A.11) $$\begin{array}{c} v_{\text{opt}}=f_{\text{opt}}=0.2653,\\ p_{\max\;\text{muscle}}=f_{\text{opt}}v_{\text{opt}}=0.07. \end{array}$$

The effects of temperature on *V*_0_ and *P*_max⁡  muscle_ in amphibian and mammalian muscles were mainly studied for values largely lower than physiological temperatures in men. The Q_10_ of *V*_0_ and *P*_max⁡_ are important between 5 and 25°C but much lower at temperature beyond 30°C [16, 218]. In slow muscle fibers, maximal power ranges between 0.3 (human, type I, 12.5°C) [18], 60 (mouse soleus, 30°C) [277], and 106 W·L^−1^ (rat soleus, 30°C) [278]. In fast muscle fibers, maximal power ranges between 1.5 (human, type IIa, 12.5°C) [18], 4.2 (human, type IIx, 12.5°C) [18], 100 (mouse extensor digitorum longus, 30°C) [277], 136 (rat gastrocnemius, 30°C) [278], and 230 W·L^−1^ (rat flexor hallucis brevis, 35°C) [218]. Moreover, the deleterious effects of acidosis and phosphate ions depend on temperature [19]. At a temperature (30°C) close to physiological temperatures, high P_i_ has no effect on *V*_0_ but reduces maximal power of the skinned fibers by depressing force and lowering the *a*/*F*_0_ ratio [19]. In addition, low pH depresses maximal power in slow and fast skinned fibers: at low temperature (15°C) the fast fibers are more sensitive to a pH decrease, whereas at a temperature (30°C) close to physiological temperatures the depression of maximal power is greater in slow fibers [19]. On the other hand, high temperature improves maximal power of skinned fibers by increasing not only *V*_0_ and *F*_0_ but also the *a*/*F*_0_ ratio. The deleterious effects of acidosis and P_i_ on muscle force are also explained by the decrease in Ca^++^ sensitivity they induce [19]. In contrast, a rise in temperature improves Ca^++^ sensitivity. The published data about *V*_0_, *F*_0_, and *a*/*F*_0_ measured at different temperatures suggest that the differences in maximal velocity of shortening and power output between fast and slow fibers at physiological temperatures are probably much less important than at low temperature (Figure 15). For example, the maximal shortening velocities at 30°C in slow fibers versus fast fibers were 4.3 versus 7.1 fl·s^−1^ [279] or 5.2 versus 6.13 fl·s^−1^ [278].

### B.

#### B.1. Biomechanics of Submaximal Cycling Exercises

Cycling is a double task: (a) moving the leg segments in such a way that the tip of foot moves on a circular trajectory; (b) producing power at the crank levels. In 2D models (sagittal plane), the cycling leg is often simplified as a system with two degrees of freedom whose actuators are mono- and biarticular muscles. Griffié and Monod [280] and Houtz and Fischer [281] who recorded the activities of many muscle groups with surface electromyograms during cycling exercises observed

1. that most of the monoarticular muscles activated during downstroke [gluteus maximus (GMax), vastus lateralis (VL), vastus medialis (VM), tibialis anterior (TA), and soleus (SOL)] are recruited in consistent orderly patterns;
2. that the patterns of recruitment of biarticular muscles [rectus femoris, semimembranosus (SM), semitendinosus (ST), the long head of the biceps femoris (BF), gastrocnemius lateralis (GL), and gastrocnemius medialis (GM)] and/or the muscle activated during upstroke or the transition phase (Top Dead Center or Bottom Dead Center) are more variable. These results were confirmed in the more recent studies on submaximal [282–287] and maximal cycling [32, 35]. The onsets (beginning) and offsets (end) of the activities of the biarticular hamstrings muscles (BF, SM, and ST) are more variable. In addition, two distinct bursts of activation have been described for BF, TA, GL, and SOL [283, 285–287]. The contribution of the deep components of the hip flexors (psoas), knee extensors (vastus medialis), and plantar ankle flexors (tibialis posterior, flexor digitorum longus) can be studied by magnetic resonance activity level [213, 285].

The muscles that participate in cycling can be gathered in four functional groups [35, 288]: (a) the uniarticular hip and knee extensors muscles (EXT); (b) the plantar flexor muscles and the biarticular hip extensors (hamstring); (c) the uniarticular hip and knee flexors (FLEX); (d) the ankle dorsiflexors (tibialis anterior) and the biarticular hip flexors (rectus femoris). These four muscle groups could be associated in two alternating pairs [35, 288]: (1) pair A-C which provides the energy during downstroke (EXT) and upstroke (FLEX); (2) pair B-D which facilitates the transition at the top (D) and bottom (B) dead centers. Submaximal cycling is the expression of three synergies corresponding to the functional muscle groups A, B, and D [37]. Rhythmic motor activity in animals is produced in large part by the activity of Central Pattern Generators (CPG) located in the spinal cord which can produce a variety of locomotor rhythms and patterns [289]. It has been suggested that a similar organization could also operate in humans, and common mechanisms of neural control could be active across many different rhythmic limb movements [205, 206]. Indeed, several studies indicate that shared circuitry could exist in humans and should be seen as a “common core” of CPG elements activated regardless of the specific locomotor task [206].

Biarticular muscles (RF, BF_*LH*⁡_, SM, ST, GM, GL) enable the energy transfer between joints. In cycling, the phases of muscle force production coincide with the phases of muscle shortening both for mono- and biarticular muscles [290]. At the end of knee extension, approximately between 100 and 170°, the net knee torque is flexing. However, during this phase, the vasti are coactivated with their biarticular antagonists, the hamstring muscles. Both monoarticular knee extensors and biarticular knee flexors exert force while shortening and, therefore, produce positive work during the end of knee extension [290]. The combination of hip and knee extensions results in a lower shortening velocity of the hamstring muscle and, consequently, higher force production for the same activation. Van Ingen Schenau et al. proposed the hypothesis that monoarticular muscles are primarily activated when they are in the position to shorten and thus to contribute to positive work, whereas biarticular muscles would control the desired direction of the external force on the pedal [291]. Most of the power produced by the hip and knee extensors is transmitted to the foot at the ankle joint, but there is a part of the quadriceps power output transferred to the foot by the gastrocnemii and the Achilles tendon during knee extension. The higher the force of the gastrocnemii, the higher the quadriceps power output which can be transmitted by the Achilles tendon.

The work produced by the muscles (*W*_muscle_) is not only transformed in work at the crank level (*W*_crank_) but is also used to move the leg segments (*W*_muscle  segment_) and increase their mechanical energy Δ*E*_segment_, that is, the sum of potential and kinetic energy (Δ*E*_segment_ = Δ*E*_potential  segment_ + Δ*E*_kinetic  segment_). The production of *W*_muscle  segment_ is not an energy waste. Indeed, according to the principle of energy conservation, there are transformations between kinetic and potential energies and energy transfer between the legs and the cycle ergometer (crank and saddle) within a pedal revolution if there is no dissipative force. Possible dissipative forces are frictional forces at the joints (ankle, knee, and foot) and the forces exerted by the active muscles which contract eccentrically. Frictional forces at joints are considered as negligible in healthy subjects. The muscles exert dissipative force when they are stretched but only when this stretching occurs while they are activated. Eccentric contractions can occur because of simultaneous activations of the antagonist muscles and/or an insufficient relaxation rate during alternating movements [41, 43]. The results of studies combining electromyography and the computation of the length muscles from movement analysis [290, 291] suggest that energy dissipation due to eccentric contraction is low during cycling exercises at low and medium pedal rates.

According to the principle of energy conservation *W*_muscles_, *W*_ergometer_, *W*_crank_, *W*_saddle_, and Δ*E*_leg_ are related according to the following equations:

(B.1) Wmuscles=Wergometer+ΔELeg=(Wcrank+Wsaddle)+ΔELeg.

If *W*_saddle_ is negligible,

(B.2) $$\begin{array}{c} W_{\text{muscles}}=W_{\text{crank}}+\Delta E_{\text{Leg}},\\ \frac{{dW}_{\text{muscles}}}{dt}=P_{\text{crank}}+\frac{{dE}_{\text{Leg}}}{dt},\\ P_{\text{muscles}}=T_{\text{crank}}\omega_{\text{crank}}+\frac{{dE}_{\text{Leg}}}{dt}, \end{array}$$

where *ω*_crank_ is crank angular velocity at time *t*, *P*_muscles_, *T*_crank_, and *P*_crank_ are the muscle power, torque, and power exerted on the crank at that time, respectively. Therefore, the instantaneous values of torque *T*_crank_ or power *P*_crank_ also depend on the transfer of energy between the leg and the crank. During upstroke, the torque measured at the crank is not the only result of leg flexor contractions but is also the result of the transfer of energy from the crank to the ascending leg. A negative torque is generally measured at the end of pedal revolution, between 240 and 360° because the subjects do not pull on the crank or the activity of the leg flexors is not strong enough. Similarly, at the end of the downstroke, the mechanical energy of the leg is transformed in external work and torque. Left and right cranks are linked together by a rigid axle, which facilitate the energy transfer between the descending and ascending legs. In a first approximation, the variations in mechanical energies of the extending and flexing legs are in phase opposition. As a consequence, the instantaneous variations in mechanical energy (kinetic and potential energies) of both legs are relatively small if the energy of the right leg is added to the left one.

In submaximal cycling at low pedal rate, the use of the inverse dynamic technique has shown that most of the power during downstroke is produced at the knee [31].

### C.

#### C.1. Estimations of *F*_0_, *V*_0_, and *P*_max⁡_ in the Study by Dickinson [109]

In 1928, Dickinson [109] published a study designed to verify Hill's hypothesis that the average external force exerted during a muscular movement, carried out with maximal effort, may be regarded as equal to a constant theoretical force diminished by an amount proportional to the speed of movement. The observed relationship between the force exerted on the pedal (*F*_*P*_) and the time *t*  of one foot movement (i.e., half a complete revolution of the crank) was

(C.1) $$\begin{array}{c} F_{P}=F_{P0}\left(1-\frac{\alpha }{t}\right)=F_{P0}-\frac{F_{P0}\alpha }{t}, \end{array}$$

where *F*_*P*0_ and *α* were constants. *F*_*P*0_ represented the maximal force (averaged over one revolution) that could be exerted at right angle to the pedal crank and attained only if the movement could take place “infinitely slowly” while *α* represented the shortest time in which the movement could be completed and attained only if no external work was done. The value of *F*_*P*0_ *α*/*t* corresponded to the amount of force proportional to the speed of movement and was attributed to internal frictional resistance in the muscles according to Hill. The inverses of *t*  and *α*  were equivalents to half pedal rate (1/*t* = 2*V*) and maximal half pedal rate (1/*α* = 2*V*_0_), and, consequently, ratio *α*/*t* was equivalent to ratio *V*/*V*_0_:

(C.2) $$\begin{array}{c} \frac{\alpha }{t}=\frac{\left(1/V_{0}\right)}{\left(1/V\right)}=\frac{V}{V_{0}},\\ F_{P}=F_{P0}\left(1-\frac{\alpha }{t}\right)=F_{P0}\left(1-\frac{V}{V_{0}}\right). \end{array}$$

This study was performed on a friction-braked ergometer (Martin's ergometer). The meter of development (*D*), that is, the distance travelled by a point of the rim for each pedal revolution, was 4.2 m instead of 6.1 m for a Monark ergometer, and the crank length was 0.18 m. The tangential force *F*_*P*_ exerted on the pedal of the Martin's ergometer corresponded to 3.85*F*, the braking force exerted on the flywheel of a Monark ergometer.

The values of *α*  ranged between 0.159 and 0.162 second which is equivalent to *V*_0_ between 189 and 177 rpm, respectively. The values of *F*_*P*0_ lay between 75 and 85 kg in 3 male subjects, which was equivalent to *F*_0_ between 19.5 and 22.1 kg for a Monark ergometer. In the 4 female subjects, *F*_*P*0_ lay between 37 and 56 kg which was equivalent to *F*_0_ between 9.6 and 14.6 kg. The individual values of *F*_0_, *V*_0_, and *P*_max⁡_ of 3 subjects in Dickinson's study were

H.D.D. (male, BM = 84.5 kg, BH = 1.76 m): *F*_0_ = 21.58 kg; *V*_0_ = 187 rpm; *P*_max⁡_ = 1009 W or 11.9 W·kg BM^−1^,
S.D. (female, BM = 53.5 kg, BH = 1.60 m): *F*_0_ = 13.52 kg; *V*_0_ = 189 rpm; *P*_max⁡_ = 639 W or 11.9 W·kg BM^−1^, and
D.H. (female; BM = 53.5 kg; BH = 1.75 m): *F*_0_ = 9.75 kg; *V*_0_ = 187 rpm; *P*_max⁡_ = 456 W or 8.5 W·kg BM^−1^.

The values of *V*_0_ were low in spite of the use of toe clips, which could be the result of the inaccuracy of the devices used in Dickinson's study [109] and/or the expression of the individual characteristics of the subjects (age? practice of endurance activities?).
